# Lysine crotonylation acts as an epigenetic switch for glutamate neurotransmission and spatial memory

**DOI:** 10.1073/pnas.2608460123

**Published:** 2026-10-28

**Authors:** Utsav Mukherjee, Budhaditya Basu, Pratyush Suryavanshi, Stacy E. Beyer, Saaman Ghodsi, Nathan Robillard, Yann Vanrobaeys, Eric B. Taylor, Joseph Glykys, Ted Abel, Snehajyoti Chatterjee

**Affiliations:** ^a^ Department of Neuroscience and Pharmacology, Carver College of Medicine, University of Iowa, Iowa City, IA 52242; ^b^ Iowa Neuroscience Institute, University of Iowa, Iowa City, IA 52242; ^c^ Interdisciplinary Graduate Program in Neuroscience, University of Iowa, Iowa City, IA 52242; ^d^ Stead Family Department of Pediatrics, Carver College of Medicine, University of Iowa, Iowa City, IA 52242; ^e^ Interdisciplinary Graduate Program in Genetics, University of Iowa, Iowa City, IA 52242; ^f^ Fraternal Order of Eagles Diabetes Research Center, University of Iowa, Iowa City, IA 52242; ^g^ Department of Molecular Physiology and Biophysics, Carver College of Medicine, University of Iowa, Iowa City, IA 52242

**Keywords:** epigenetics, memory, chromatin, gene expression, glutamate

## Abstract

Histone posttranslational modifications, particularly lysine acetylation (Kac), are critical epigenetic regulators of transcriptional programs underlying long-term memory consolidation. Beyond Kac, several other non-acetyl acylations have been identified with the ability to regulate transcription; however, their role in memory consolidation remains unknown. Here, we identify histone lysine crotonylation (Kcr) as a molecular switch for long-term memory and glutamatergic neurotransmission. We find that spatial learning induces distinct spatiotemporal patterns of Kcr across hippocampal subregions. Through genetic manipulations, we show that reducing hippocampal Kcr levels impairs memory, while increasing Kcr levels by means of crotonate administration enhances long-term memory. Single-nuclei transcriptome and chromatin accessibility analyses reveal that crotonate facilitates activation of genes regulating glutamate signaling. Cell–cell communication analysis further suggests a strengthening of glutamatergic signaling within hippocampal excitatory neurons following crotonate administration. Real-time fluorescence imaging with genetically encoded sensors functionally validates our multiomics and computational findings—demonstrating the role of Kcr in regulating presynaptic glutamate release and neuronal activity. In summary, our findings elucidate a key mechanism underlying long-term memory consolidation and excitatory neurotransmission, linking epigenetic events to synaptic function and behavior.

## Significance

Although histone acetylation has long been considered a principal regulator of gene expression events underlying memory storage, the role of non-acetyl histone acyl modifications on long-term memory remains unknown. Here, by integrating pharmacological, genetic, transcriptomic, epigenomic, and real-time imaging approaches, we identify histone lysine crotonylation as a key epigenetic regulator of hippocampal memory consolidation and glutamatergic signaling. These findings expand the conceptual framework of epigenetic control of long-term memory storage by demonstrating the functional relevance of non-acetyl histone acylations and lays the foundation for targeting this epigenetic mark to combat cognitive decline in neurological and neuropsychiatric disorders.

Histone posttranslational modifications (PTMs) play a key role in the epigenetic regulation of gene expression during memory consolidation (1–5). Short-chain acyl modifications on histone lysine residues are well-known PTMs that regulate several biological processes (6–8). Among these, histone lysine acetylation (9) has been extensively studied for its role in memory storage. Altering histone Kac levels by selectively manipulating the “writers” (lysine acetyltransferases, KATs) and “erasers” (lysine deacetylases, KDACs) of histone Kac has been shown to impact hippocampal memory storage (2, 10, 11). Interestingly, studies have revealed the promiscuous nature of the majority of the writers and erasers for histone Kac—demonstrating that these enzymes can also catalyze the incorporation or removal of non-acetyl acyl moieties on histone lysine residues (6, 9, 12). This raises the question of whether the impact of manipulating KATs and KDACs on memory consolidation, as demonstrated in previous studies (13–18), could be attributed to underlying changes in non-acetyl histone acylations besides Kac. Elucidating the functional relevance of non-acetyl histone acylations in long-term memory is therefore essential for advancing our conceptual understanding of epigenetic mechanisms underlying memory consolidation.

Advancements in mass spectrometry and biochemical approaches have led to the identification of several novel histone lysine acylations that are structurally distinct from histone Kac (19, 20). Among these, histone lysine crotonylation (Kcr), an evolutionarily conserved non-acetyl acylation, has gained significant attention in recent years for its role in transcription regulation (20–26). Kcr is enriched on active gene promoters (20), and Kcr-mediated regulation of gene expression has been implicated in diverse biological functions, including DNA damage repair (21), acute kidney injury (25), spermatogenesis (22), nerve-injury-induced-neuropathic pain (26), and neural stem cell differentiation (23). Interestingly, studies in cell-free systems have demonstrated that the lysine acyltransferase p300 stimulates histone Kcr-mediated transcription to a significantly higher magnitude than histone Kac (12). Furthermore, several histone “reader” proteins—initially characterized as Kac readers—exhibit a higher binding affinity for crotonylated lysine residues compared to acetylated lysine moieties, and this preferential binding is thought to underlie the ability of the readers to regulate transcription (27). We and others have shown that precisely timed transcriptional events, induced after learning, are critical for long-term memory consolidation (28–31). Although Kcr has been well established as a transcriptional activator, its physiological relevance to memory consolidation and the Kcr-dependent gene expression programs that underlie hippocampal memory storage remain elusive.

The availability of metabolic precursors dictates the landscape of histone acylations (6). Accordingly, changes in intracellular crotonyl-CoA levels have been shown to alter histone Kcr levels, with direct consequences on gene transcription. Increasing crotonyl-CoA levels through crotonate administration elevates Kcr and stimulates gene transcription (12, 23), whereas depletion of Kcr via manipulation of the crotonyl-CoA hydratase chromodomain Y-like protein (CDYL) represses transcription (24). Interestingly, in addition to writers and erasers, histone Kac and histone Kcr share enzymes that synthesize their respective metabolic precursors. The enzyme Acetyl-CoA synthetase 2 (ACSS2) synthesizes both acetyl CoA and crotonyl-CoA (12, 32). We previously demonstrated that ACSS2 is a critical regulator of neuronal histone Kac and spatial memory consolidation in the hippocampus (33). Silencing ACSS2 levels reduces histone Kac and impairs activity-dependent transcription of memory-related genes (33). Notably, knockdown of ACSS2 has also been shown to reduce Kcr levels and repress Kcr-regulated inflammatory gene transcription (12). However, whether ACSS2-mediated regulation of histone Kcr constitutes a critical molecular mechanism underlying long-term memory consolidation remains unknown. Altogether, these findings raise the possibility that metabolic coupling of crotonyl-CoA availability to Kcr-dependent chromatin plasticity represents a critical mechanism linking neuronal metabolism to memory storage and learning-induced transcriptional events.

Here, we provide evidence demonstrating the role of Kcr as an epigenetic rheostat for hippocampal memory consolidation and glutamatergic neurotransmission. We establish crotonate as a pharmacological agent that increases histone Kcr levels in the dorsal hippocampus and enhances long-term memory. We then dissect the molecular underpinnings of crotonate administration during the early critical window of memory consolidation, revealing its impact on transcriptional activation of genes regulating synaptic function and glutamatergic neurotransmission. Finally, we elucidate the functional consequences of crotonate on glutamatergic neurotransmission and neuronal activity at the circuit level. Together, our findings reveal a role for a non-acetyl acyl modification in long-term memory and excitatory neurotransmission. More importantly, our study provides molecular insights into how epigenetic tuning of transcriptional programs by histone PTMs translate into changes in neuronal function and behavioral outcomes—linking nuclear epigenetic events to synaptic function.

## Results

### Spatial Training Induces Lysine Crotonylation in Discrete Hippocampal Subregions.

**Fig. 1.**
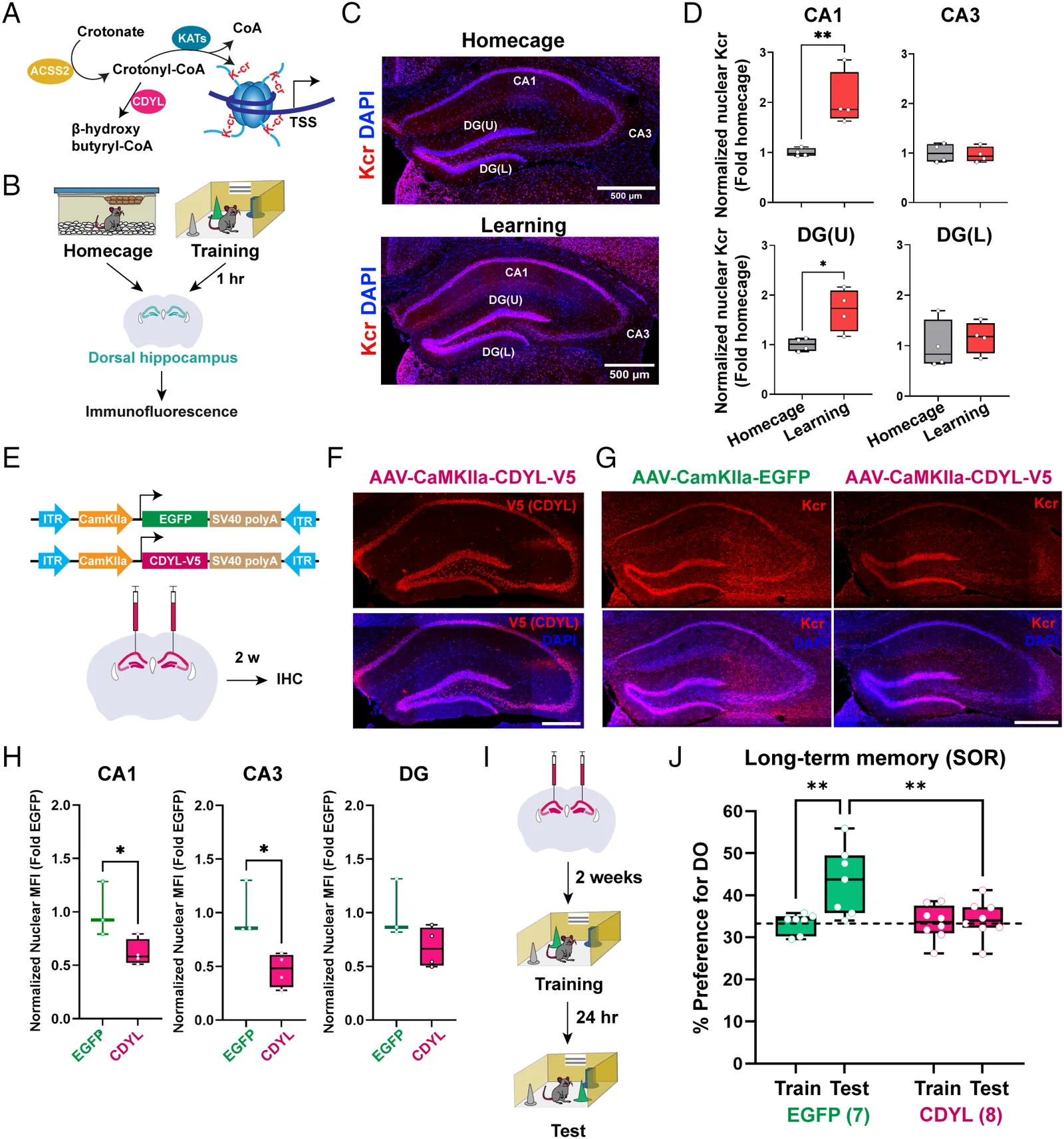
Lysine crotonylation is a critical regulator of long-term spatial memory consolidation. (*A*) Depiction of molecular regulators of histone lysine crotonylation (Kcr). (*B*) Schematic of the experiments performed in *C* and *D*. (*C*) Immunofluorescence using anti-Kcr antibody showing levels of Kcr in different hippocampal subregions of HC and learning (SOR +1 h) mice. (*D*) Normalized Mean Fluorescent Intensity (MFI) of nuclear Kcr levels across the groups. For CA1: unpaired *t* test: t (6) = 3.810, **P = 0.0089. Homecage (n = 4) and SOR+1 h (n = 4), males only. For CA3: unpaired *t* test: t (6) = 0.2749, *P* = 0.7926. Homecage (n = 4) and SOR + 1 h (n = 4), males only. For DG upper blade (U): unpaired *t* test: t (6) = 3.118, *P = 0.0206. Homecage (n = 4) and SOR +1 h (n = 4), males only. For DG lower blade (L): unpaired *t* test: t (6) = 0.5452, *P* = 0.605. Homecage (n = 4) and SOR +1 h (n = 4), males only. (*E*) Schematic of viral constructs transduced in the dorsal hippocampus of adult male mice to overexpress Chromodomain Y-like protein (CDYL). AAV_9_-CaMKIIα-EGFP served as vector control, while AAV_9_-CaMKIIα-CDYL-V5 was used to drive expression of CDYL in excitatory neurons. (*F*) Immunofluorescence using V5 antibody showing ectopic expression of CDYL-V5 in dorsal hippocampus 2 wk after viral infusion. (*G*) Immunofluorescence image of the dorsal hippocampus using pan-Kcr antibody in CDYL-V5 or control expressing mice. (*H*) Normalized Mean Fluorescent Intensity (MFI) of nuclear Kcr levels across the groups. For CA1: Unpaired *t* test: t (5) = 2.679, *P = 0.0439. EGFP (n = 3) and CDYL (n = 4), males only. For CA3: Unpaired *t* test: t (5) = 3.417, *P = 0.0189. EGFP (n = 3) and CDYL (n = 4), males only. For DG: Unpaired *t* test: t (5) = 1.863, *P* = 0.1215. EGFP (n = 3) and CDYL (n = 4), males only. (*I*) Long-term memory (24 h) assessment of mice infused with AAV-EGFP or AAV-CDYL into dorsal hippocampus. (*J*) Quantification of *I*. Two-way ANOVA: significant session × virus interaction F_(1,13)_ = 6.323, *P* = 0.0259, Sidak’s multiple comparison tests, **P = 0.0041 (EGFP 24 h Test vs. CDYL 24 h Test) and **P = 0.0051 (EGFP Training vs. EGFP 24 h Test); AAV-EGFP (n = 7), AAV-CDYL (n = 8), males only. All box plots: the center line represents the median, the box edges represent the top and bottom quartiles (25th to 75th percentiles), and the minimum and maximum whiskers.

KATs catalyze the addition of crotonyl groups on histone lysine residues using crotonyl-CoA as a cofactor, and crotonyl-CoA levels are regulated by the enzymes ACSS2 and CDYL (Fig. 1*A*). We first investigated whether spatial learning induces changes in histone Kcr in the hippocampus, similar to the well-established increases in histone Kac following spatial training (2, 10, 34). We trained wild-type (WT) mice in a spatial object recognition (SOR) task and examined Kcr levels in core histones extracted from the dorsal hippocampus at different time-intervals (0.5-, 1-, and 2-h) post training (*SI Appendix*, Fig. S1 *A* and *B*). We focused on the dorsal hippocampus in all our experiments, given extensive evidence for its critical role in spatial memory consolidation (30, 35–37). Moreover, in the absence of prior knowledge regarding specific crotonylated histone lysine residues that respond to learning, we measured pan-lysine crotonylation (Kcr) to assess the global landscape of histone Kcr during spatial memory consolidation. Using an antibody that has been shown to selectively detect crotonylated lysine residues (12) and has been extensively used to characterize Kcr in several other studies (24, 38–41), we observed that histone Kcr levels significantly increased at the 1-h timepoint after SOR training and returned to baseline levels at the 2-h timepoint (*SI Appendix*, Fig. S1 *A* and *B*). Next, we performed immunofluorescence using the pan-Kcr antibody to map the spatial profiles of Kcr induction in the hippocampus following SOR training (Fig. 1 *B*–*D*). Quantitative analysis of the nuclear Kcr levels revealed significant increases in the CA1 pyramidal layer and the upper blade of dentate gyrus (DG) (Fig. 1 *C* and *D*), and in the subiculum (*SI Appendix*, Fig. S2 *A* and *B*) at the 1-h timepoint after training in the SOR task, whereas no significant changes in the nuclear Kcr levels were observed in hippocampal subregions CA3 and the lower blade of DG (Fig. 1 *C* and *D*). These results identify histone Kcr to exhibit distinct spatiotemporal dynamics in the dorsal hippocampus following spatial learning.

### Reduction of Hippocampal Kcr Levels Impairs Long-Term Spatial Memory.

The crotonyl-CoA hydratase Chromodomain Y-like protein (CDYL) hydrolyzes crotonyl-CoA to beta-hydroxy butyryl-CoA (Fig. 1*A*), thereby reducing histone Kcr levels (24). To date, CDYL remains the only bona fide negative regulator of Kcr that does not affect other histone acylations (39)—presenting us with a unique strategy to selectively manipulate histone Kcr levels and examine its impact on memory consolidation. Interestingly, neuronal activity has been shown to reduce CDYL levels (42), prompting us to investigate whether spatial learning would similarly impact hippocampal CDYL levels. We performed immunofluorescence on brain sections obtained from SOR-trained WT mice to examine spatial patterns of CDYL protein levels following learning across hippocampal subregions (*SI Appendix*, Fig. S3 *A*–*D*). Hippocampal subregions CA1, subiculum, and the upper blade of DG exhibited significant downregulation of nuclear CDYL levels 1-h after SOR training, whereas nuclear CDYL levels remained unchanged in the hippocampal subregions CA3 and the lower blade of DG (*SI Appendix*, Fig. S3 *A*–*D*). The downregulation of nuclear CDYL levels after learning in CA1, subiculum, and the upper blade of DG offers a molecular explanation for the corresponding learning-induced increase in nuclear Kcr levels observed within these hippocampal subregions.

Next, to determine whether histone Kcr has a role in hippocampal long-term memory, we designed a genetic strategy to selectively modulate Kcr in the dorsal hippocampus by manipulating levels of CDYL. We injected an adeno-associated virus in WT mice to overexpress CDYL (AAV_9_-CaMKIIα-CDYL-V5) in excitatory neurons of the dorsal hippocampus (Fig. 1*E*). Immunofluorescence performed 2 wk after viral infusion confirmed the expression of CDYL-V5 across the dorsal hippocampal principal neuronal layers (Fig. 1*F*). We observed significantly reduced Kcr levels in the hippocampal areas CA1 and CA3 in CDYL-V5-expressing mice compared to EGFP controls (Fig. 1 *G* and *H*), thus validating the efficacy of our viral-based approach. Next, we evaluated long-term spatial memory in mice infused with AAV-CDYL-V5 in the dorsal hippocampus. Mice were trained in the SOR task with objects that were placed in specific spatial locations within a SOR arena. To test for long-term spatial memory, we performed a retrieval task 24-h later with one of the objects displaced to a novel spatial location (Fig. 1*I*). Control AAV-infused mice (AAV_9_-CaMKIIα-EGFP) exhibited a higher preference toward the displaced object during the test session, suggesting intact memory consolidation. Conversely, CDYL-infused mice (AAV_9_-CaMKIIα-CDYL-V5) failed to exhibit any preference toward the displaced object during the test session (Fig. 1*J*). To examine potential confounds due to changes in anxiety-related behavior, we quantified the time spent in the inner and outer zones of the open field during habituation. Our analysis showed that both groups of mice spent an equivalent amount of time in the inner or the outer zones (*SI Appendix*, Fig. S4), thereby implying that the effects of CDYL on memory are unlikely to be attributable to changes in anxiety-related behaviors. Collectively, these findings suggest a critical role of hippocampal Kcr in spatial memory consolidation.

### Pharmacologically Increasing Kcr Enhances Long-Term Memory.

**Fig. 2.**
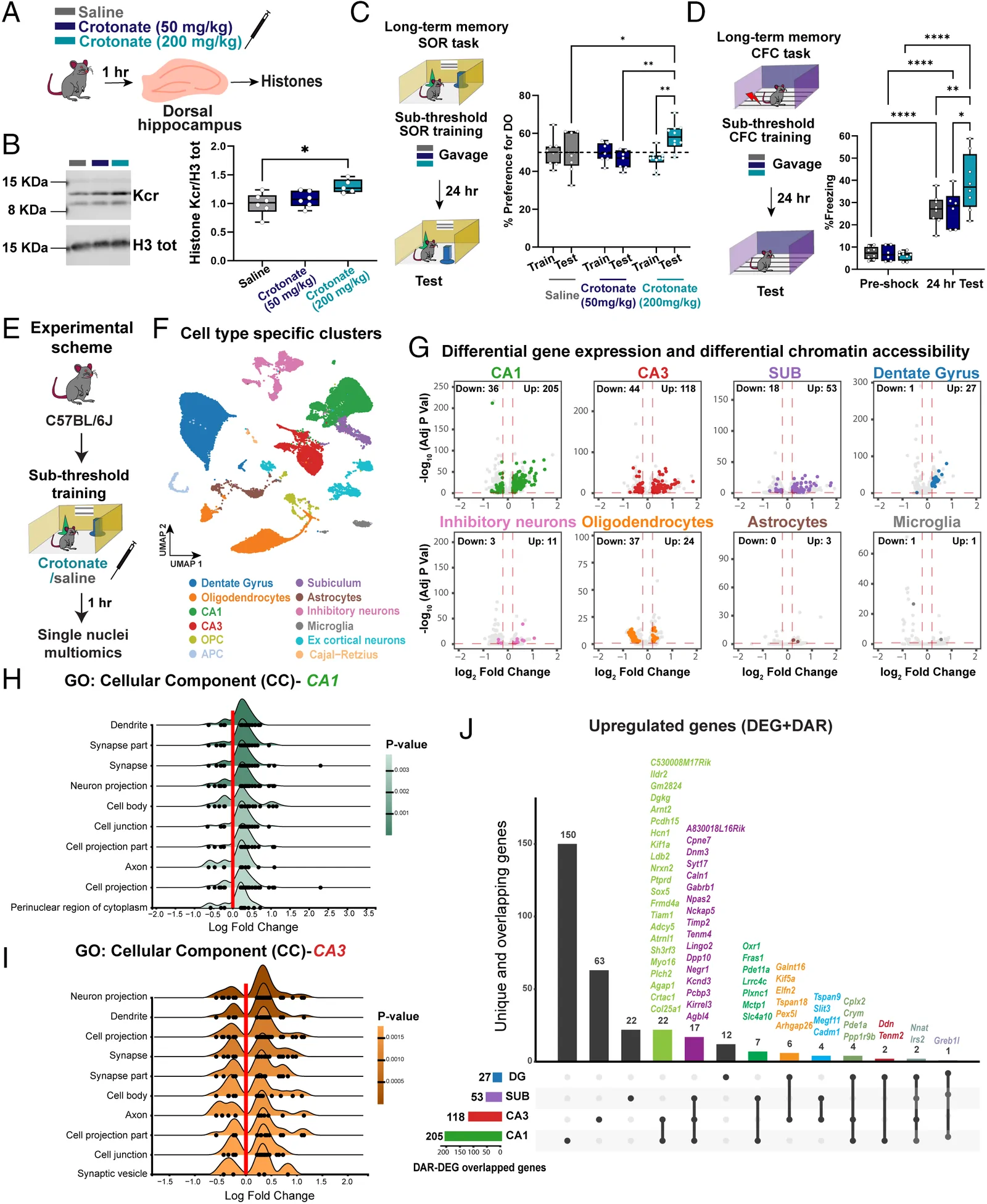
Single-nuclei multiomics (RNA + ATAC-seq) reveals mechanistic insights underlying lysine crotonylation-mediated memory enhancement. (*A*) Kcr western blots of core histones extracted from dorsal hippocampus 1 h after crotonate treatment (50 mg/kg or 200 mg/kg dose). Vehicle (saline) treated mice were used as controls. (*B*) Quantification of *A*. One-way ANOVA: Kcr: F_(2, 14)_ = 5.550, *P* = 0.0168. Dunnett’s multiple comparisons tests: *P = 0.0108 (saline vs. crotonate 200mg/kg). Saline (n = 6), Crotonate 50 mg/kg (n = 6), Crotonate 200 mg/kg (n = 5), males only. (*C*) Long-term memory enhancement in a subthreshold SOR learning paradigm following crotonate treatment. Two-way ANOVA: significant session × treatment interaction F_(2, 23)_ = 5.081, *P* = 0.0149, Sidak’s post hoc tests, *P = 0.0359 (200 mg/kg crotonate 24 h test vs. saline 24 h test), **P = 0.0021 (200 mg/kg crotonate train vs. 200mg/kg crotonate 24 h test), **P = 0.0025 (200 mg/kg crotonate 24 h test vs. 50mg/kg crotonate 24 h test). Saline (n = 9), Crotonate 50 mg/kg (n = 7), Crotonate 200 mg/kg (n = 10), males only. (*D*) Long-term memory in a subthreshold CFC learning paradigm following crotonate treatment. Two-way ANOVA: Significant session × treatment interaction F_(2, 19)_ = 4.946, *P = 0.0187, main effect of session (preshock and 24 h test): F_(1, 19)_ = 140.9, *P* < 0.0001. Sidak’s post hoc tests, *P = 0.0130 (200 mg/kg crotonate 24 h test vs. 50 mg/kg crotonate 24 h test), **P = 0.0051 (200 mg/kg crotonate 24 h test vs. saline 24 h test). ****P < 0.0001 (Saline preshock vs. saline 24 h test), ****P < 0.0001 (crotonate 50 mg/kg preshock vs. crotonate 50 mg/kg 24 h test), and ****P < 0.0001 (crotonate 200 mg/kg preshock vs. crotonate 200 mg/kg 24 h test). Saline (n = 8), Crotonate 50 mg/kg (n = 6), Crotonate 200 mg/kg (n = 8), males only. (*E*) Experimental scheme: Adult male C57BL/6J mice were trained in a subthreshold SOR paradigm and administered with crotonate (200 mg/kg, oral gavage; n = 4) or saline (oral gavage; n = 4) immediately after the completion of training. 1-h later, the dorsal hippocampus was harvested, and nuclei were isolated for single-nuclei multiomics processing. Hippocampi from two animals within the same group were pooled for each droplet capture, resulting in a final sample size of n = 2 per group for the single-nuclei multiomics analysis. (*F*) UMAP plot showing cell type–specific clusters of the dorsal hippocampus. (*G*) Volcano plots depicting genes that exhibit differential expression and chromatin accessibility following crotonate treatment. Genes labeled with color are DEGs that also exhibit DARs, while genes that just exhibit differential expression but do not exhibit DARs are labeled in gray. (*H* and *I*) Ridgeline plots depicting the top 10 enriched pathways (GO: Cellular Component) and their corresponding DEGs with DARs in hippocampal subregions CA1 (*H*) and CA3 (*I*) that are upregulated in response to crotonate administration. The Log Fold change of the RNA component from the multiomic analysis has been plotted corresponding to each pathway. (*J*) Upset plot depicting unique and overlapping population of upregulated DEGs with DARs across all the hippocampal principal neurons. All box plots: the center line represents the median, the box edges represent the top and bottom quartiles (25th to 75th percentiles), and the minimum and maximum whiskers.

Given that a reduction in hippocampal Kcr levels significantly impairs long-term memory, we next asked whether increasing Kcr levels could enhance long-term memory. Administration of crotonate, a short-chain fatty acid, has been shown to elevate histone Kcr (12, 23) and stimulate gene expression (23) by increasing Crotonyl-CoA levels (12, 23). Therefore, we implemented this strategy to pharmacologically increase histone Kcr levels in mice. We administered two different doses of crotonate (50 and 200 mg/kg) to WT mice by oral gavage and examined histone Kcr levels in the dorsal hippocampus 1-h after crotonate administration (Fig. 2*A*). We observed a significant increase in histone Kcr following 200 mg/kg dose of crotonate, whereas the 50 mg/kg dose of crotonate failed to enhance histone Kcr levels compared to vehicle (saline)-treated mice (Fig. 2*B*). To determine the specificity of crotonate treatment, we examined histone acetylation levels (pan H3-acetylation) after crotonate administration and found no changes in levels of H3 acetylation (*SI Appendix*, Fig. S5). Next, to study the impact of higher histone Kcr levels on long-term memory, we trained WT mice in a weak-learning (subthreshold) SOR paradigm (43) to evaluate long-term memory enhancement. The subthreshold variant of the SOR task was designed to assess enhancements in long-term memory, as weak SOR training protocols typically do not result in long-term memory (43–45). Mice were administered 50 mg/kg of crotonate, 200 mg/kg of crotonate, or vehicle via oral gavage immediately after the completion of the training session. When tested 24 h later, both the vehicle-treated and 50 mg/kg crotonate-treated mice did not show any preference toward the displaced object, whereas mice administered with 200 mg/kg crotonate showed a significant preference toward the displaced object (Fig. 2*C*). Consistent with our SOR findings, we observed a significantly enhanced freezing response upon crotonate administration when mice were tested 24 h following a subthreshold contextual fear conditioning (CFC) task (Fig. 2*D*). Our behavioral findings demonstrate that increases in histone Kcr augments hippocampal long-term spatial and contextual fear memories, further underscoring the functional relevance of Kcr in long-term memory consolidation.

### Neuronal ACSS2 Is a Key Regulator of Crotonate-Dependent Memory Enhancement.

The metabolic enzyme ACSS2 has been shown to synthesize acetyl-CoA. Previous work has demonstrated that ACSS2 plays a critical role in long-term memory by coupling acetyl-CoA synthesis to Kac-mediated transcription of learning-responsive genes (33). However, ACSS2 can also synthesize crotonyl-CoA from the short-chain fatty acid crotonate in mammalian cells (12) (Pathway shown in Fig. 1*A* and *SI Appendix*, Fig. S6*A*). This prompted us to investigate whether ACSS2 regulates the increases in histone Kcr and enhancements in long-term memory seen after the administration of crotonate. We injected an AAV vector (AAV_9_-CaMKIIα-Cre-EGFP) into the dorsal hippocampus of male ACSS2^f/f^ mice (46) to achieve a conditional knockdown of ACSS2 expression selectively in the excitatory neurons of the dorsal hippocampus (*SI Appendix*, Fig. S6*B*). Immunofluorescence performed on brain sections obtained from the ACSS2 conditional knockout mice (ACSS2 cKO mice; ACSS2 ^f/f^:CaMKIIα-Cre-EGFP) revealed the expression of Cre-EGFP across hippocampal subregions (*SI Appendix*, Fig. S6*C*). Western blot analyses confirmed the downregulation of ACSS2 in the dorsal hippocampus of ACSS2 cKO mice (*SI Appendix*, Fig. S6 *D* and *E*). We then examined the impact of crotonate administration on histone Kcr levels in ACSS2 cKO mice. We administered crotonate (200 mg/kg) or vehicle (saline) via oral gavage immediately after a subthreshold SOR training session, and quantified histone Kcr levels in the dorsal hippocampus 1-h after training. We found that crotonate treatment failed to enhance hippocampal histone Kcr levels in ACSS2 cKO mice (*SI Appendix*, Fig. S6 *F* and *G*), despite administering a dose of crotonate (200 mg/kg) that was sufficient to induce histone Kcr levels in WT mice (Fig. 2*B*). As crotonate administration failed to enhance Kcr levels in ACSS2 cKO mice, we hypothesized that crotonate treatment would also fail to elicit long-term memory enhancement in ACSS2 cKO mice. To test this hypothesis, we trained ACSS2 cKO mice in a subthreshold learning SOR paradigm, administered vehicle or crotonate (200 mg/kg) immediately after training, and examined their long-term memory 24 h after training (*SI Appendix*, Fig. S6*H*). Our behavioral assessment confirmed that crotonate treatment in ACSS2 cKO mice had no impact in long-term memory compared to the vehicle-treated group (*SI Appendix*, Fig. S6*I*). Taken together, these results demonstrate that ACSS2 is critical in mediating the molecular impact of crotonate-mediated increases in Kcr on memory consolidation.

### Single-Nuclei Multiomics Reveals the Molecular Impact of Crotonate in Hippocampal Cell Types During Memory Consolidation.

Next, we implemented a single-nuclei multiomics (snRNA-seq and snATAC-seq) strategy to elucidate the molecular impact of crotonate during the early critical window of memory consolidation. Mice were trained in SOR using a subthreshold learning paradigm, and oral administration of crotonate (200 mg/kg) or vehicle (saline) was performed immediately after training. 1-h after crotonate administration, the dorsal hippocampus was harvested, and nuclei were isolated for single-nuclei multiomics (Fig. 2*E*). Cell type clustering identified well-distinguished clusters comprising excitatory and inhibitory neurons, as well as clusters representing non-neuronal cell populations (Fig. 2*F*). Excitatory neurons were further classified into CA1, CA3, subiculum (Sub), dentate gyrus, and excitatory cortical neurons based on cell-type-specific marker gene expression (Fig. 2*F* and *SI Appendix*, Fig. S7*A*). Notably, crotonate and vehicle treatment groups exhibited the same number of cell-type-specific clusters and comparable proportion of cell-types (*SI Appendix*, Fig. S7 *B* and *C* and Dataset S1). Next, we performed differential gene expression (DEG) utilizing the snRNA-seq data along with differential accessibility regions on the chromatin from the snATAC-seq data of each cluster, comparing crotonate and vehicle treated groups. For downstream analysis, we used the DEGs (Upregulated: FDR < 0.05, absolute log_2_ fold change > 0.2; Downregulated: FDR<0.05, absolute log_2_ fold change < −0.2) exhibiting concordant DARs (Upregulated: FDR < 0.05, absolute log_2_ fold change > 0.2; Downregulated: FDR<0.05, absolute log_2_ fold change < −0.2) across their promoter and gene body (Fig. 2*G* and Dataset S2). We found that excitatory neurons in the CA1 hippocampal area showed the highest number of genes impacted by crotonate, with 205 upregulated genes showing increased chromatin accessibility and 36 downregulated genes showing reduced chromatin accessibility (Fig. 2*G*). Other cell types that showed a strong impact of crotonate on gene expression and chromatin accessibility were CA3 excitatory neurons (118 upregulated genes with increased chromatin accessibility and 44 downregulated genes with reduced chromatin accessibility), subiculum (53 upregulated genes with increased chromatin accessibility and 18 downregulated genes with reduced chromatin accessibility), and the DG (27 upregulated genes with increased chromatin accessibility and one downregulated gene with reduced chromatin accessibility) (Fig. 2*G*). In addition to the principal neuronal clusters (CA1, CA3, subiculum, and DG), our analysis identified discrete molecular changes in inhibitory neurons (11 upregulated genes with increased DARs and 3 downregulated genes with reduced DARs) (Fig. 2*G*). Among the non-neuronal cells, oligodendrocytes showed the highest number of overlapping DEGs and DARs (24 upregulated and 27 downregulated) (Fig. 2*G*). Notably, we found a significant correlation between the significant differentially expressed genes and genes with significant differential chromatin accessibility in hippocampal subregions CA1 and CA3 (*SI Appendix*, Fig. S8).

To better understand the impact of crotonate on the molecular signatures of principal neuronal subtypes in the dorsal hippocampus, we performed Gene Ontology (GO: Cellular Component) overrepresentation analysis to determine the subcellular localization of the most enriched pathways among DEGs associated with DARs following crotonate treatment (Fig. 2 *H* and *I* and Dataset S3). Notably, we found that crotonate administration led to changes in chromatin accessibility and transcription of several genes that encode for proteins localized at synaptic compartments in both CA1 and CA3, as evidenced by the enrichment of the GO terms “dendrite,” “synapse,” “synapse part,” and “cell junction” (Fig. 2 *H* and *I* and Dataset S3). We also performed a GO overrepresentation analysis based on Molecular Function (GO: Molecular Function) to characterize the functionality of the pathways enriched among the DEGs with DARs following crotonate treatment (*SI Appendix*, Fig. S9). Among the most significant pathways found enriched, cell adhesion molecule binding was commonly enriched in subregions CA1, CA3, and subiculum, whereas distinct pathways attributed to ion channel activity were commonly enriched in CA1 and CA3 (*SI Appendix*, Fig. S9 and Dataset S4). In contrast, pathways attributed to PDZ domain binding and calcium-dependent phospholipid binding were found enriched in the subiculum, whereas DG exhibited an enrichment of pathways related to protein kinase C activity and serine/threonine kinase activity. (*SI Appendix*, Fig. S9). Among the downregulated DEGs with reduced chromatin accessibility, we found pathways attributed to ephrin receptor activity, calcium ion transmembrane transporter activity, and phosphoric diester hydrolase activity downregulated in CA3, whereas pathways involved in cadherin binding and beta-catenin binding were downregulated in DG (*SI Appendix*, Fig. S10).

Next, utilizing an Upset plot, we compared unique and overlapping upregulated genes with concordant DARs in each cell-type. We found that CA1, CA3, subiculum, and DG showed a common upregulation of 2 genes (Fig. 2*J*). Notably, CA1, CA3, and subiculum showed 17 commonly upregulated genes, CA1, CA3, and DG showed a common upregulation of four genes, and CA1 and CA3 showed 22 commonly upregulated genes following crotonate treatment (Fig. 2*J*). Like the cell-type expression patterns observed with upregulated DEGs with increased DARs, there was some overlap in the expression of downregulated genes with reduced DARs between cell-types. We identified 23 downregulated genes specific to CA1, 32 exclusively in CA3, and 12 selectively in the subiculum (*SI Appendix*, Fig. S11). Four genes were commonly downregulated across CA1, CA3, and the subiculum, while 8 downregulated genes were shared between CA1 and CA3 (*SI Appendix*, Fig. S11). Because the manipulation of Kcr levels selectively in excitatory neurons was found to impact long-term memory (Fig. 1 *E*–*G* and *SI Appendix*, Fig. S4), our subsequent analyses focused on DEGs within the neuronal populations of CA1, CA3, subiculum, and DG that also exhibit DARs.

To further gain an understanding of how crotonate-mediated changes in chromatin accessibility activate gene transcription, we performed a Transcription Factor (TF) Motif Enrichment analysis on the promoters of the upregulated genes that exhibit increased chromatin accessibility following crotonate administration (*SI Appendix*, Fig. S12 and Dataset S5). Among the top 15 most significant TF motifs enriched on the promoters of upregulated DEGs across the cell clusters, TF motifs KLF15, ZNF148, and MAZ were found commonly enriched across subregions CA1, CA3, and subiculum. Commonalities in the enrichment of TF motifs on upregulated DEG promoters were observed across hippocampal subregions, such as EGR1 and CTCFL in CA1 and CA3. No significant TF motifs were found enriched in the upregulated DEGs in the DG.

### Cell–Cell Communication Analysis Computes Enhanced Hippcampal Glutamate Signaling Following Crotonate Administration.

**Fig. 3.**
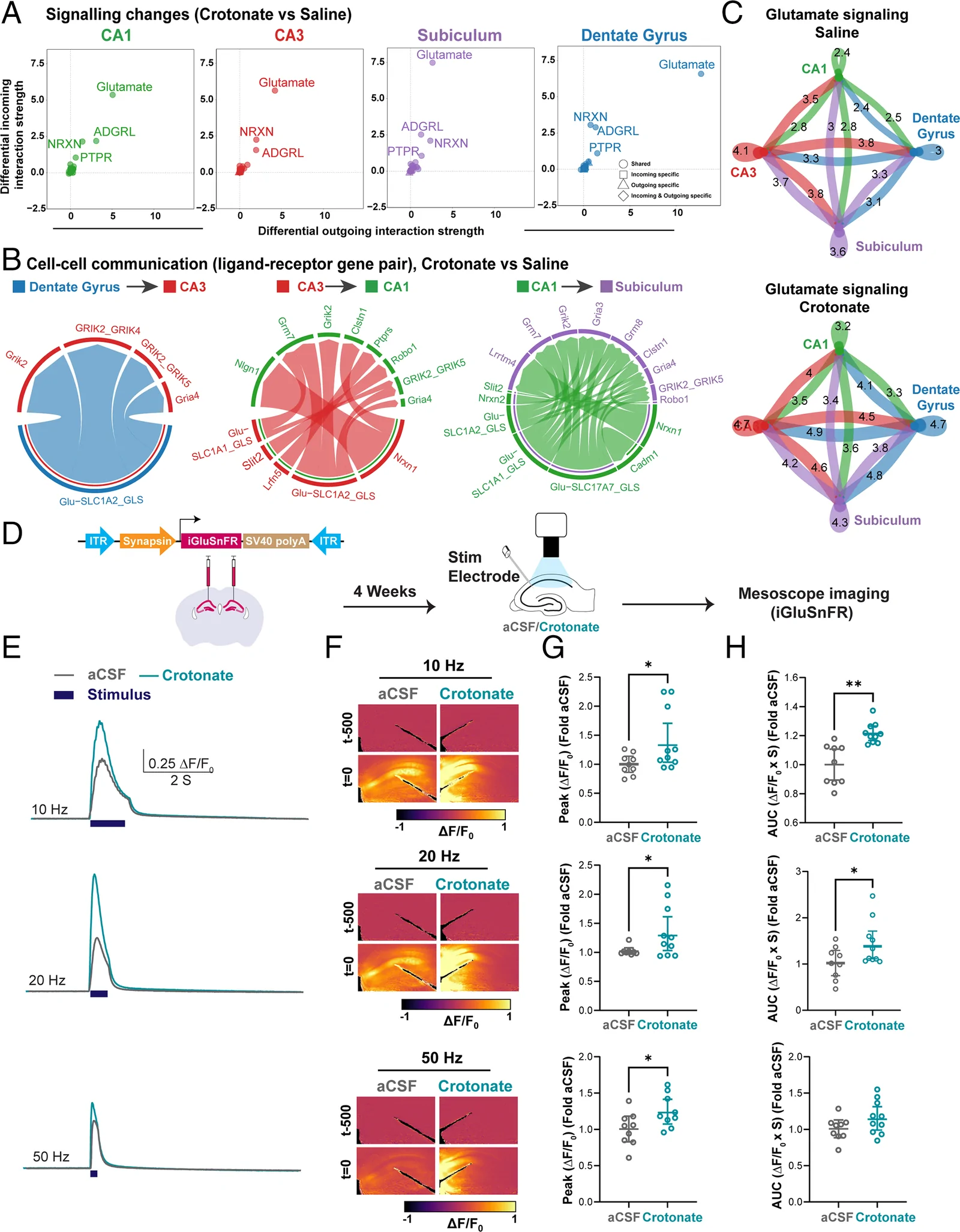
Crotonate-mediated increases in Kcr augments glutamatergic neurotransmission. (*A*) Differential incoming interaction strength and differential outgoing interaction strength in crotonate-treated mice compared to saline-treated mice in hippocampal subregions CA1, CA3, Subiculum, and DG. Differential changes in interaction strength in “Shared” (both incoming and outgoing connections in both the groups), “Incoming specific” (incoming connections in both the groups), “Outgoing specific” (outgoing connections in both the groups), and “Incoming and Outgoing specific” (both incoming and outgoing connections in either of the groups) clusters are depicted by symbols. (*B*) Individual ligand–receptor interactions significantly enhanced upon crotonate treatment in intrahippocampal projections of DG-CA3, CA3-CA1, and CA1-subiculum. The width of the edges corresponding to each ligand–receptor pair indicates the computed strength of their communication. (*C*) Interaction strength of ligand–receptor genes involved in glutamatergic signaling across the principal hippocampal neuronal networks in saline-treated and crotonate-treated mice. Communication probability, represented by numbers ranging from 0 to 10, is visualized through the edge width of intrahippocampal connections, indicating the strength of communication between the respective subregions. (*D*–*H*) Kcr enhancement leads to larger stimulus-evoked glutamate transients in acute hippocampal slices. (*D*) Schematic of the experimental design. (*E*) Representative traces of evoked glutamate transient responses at 10, 20, and 50 Hz in control slices and ones incubated with crotonate (5mM). (*F*) Image panels showing stimulus-evoked glutamate transient response over time in the hippocampus upon stimulating the Schaffer collaterals in the CA1 Stratum Radiatum. The black object in each image depicts the stimulating electrode. t = 0 denotes the time point corresponding to the peak amplitude; t − 500 represents the time point 500 ms prior to t = 0. (*G*) The peak amplitude of evoked glutamate transients was significantly higher in crotonate-treated slices compared to control across all the stimulation frequencies. Normalized Peak Amplitude across the groups. For 10 Hz: Lognormal Welch’s *t* test: t (13.96) = 2.37, *P = 0.0327. aCSF (n = 9) and Crotonate (n = 10). For 20 Hz (*Middle*): Lognormal Welch’s *t* test: t (9.982) = 2.302, *P = 0.0442. aCSF (n = 9) and Crotonate (n = 10). For 50 Hz (*Bottom*): Lognormal Welch’s *t* test: t (14.72) = 2.274, *P = 0.0384. aCSF (n = 9) and Crotonate (n = 10). (*H*) The area under the curve of evoked glutamate transients was significantly higher in crotonate-treated slices compared to control in the 10 Hz and 20 Hz stimulation groups. For 10 Hz: Lognormal Welch’s *t* test: t (10.34) = 4.043, **P = 0.0022. aCSF (n = 9) and Crotonate (n = 10). For 20 Hz (*Middle*): Lognormal Welch’s *t* test: t (14.96) = 2.263, *P = 0.039. aCSF (n = 9) and Crotonate (n = 10). For 50 Hz (*Bottom*): Lognormal Welch’s *t* test: *t* (16.92) = 1.625, *P* = 0.1226. aCSF (n = 9) and Crotonate (n = 10). All scatter plots: the center line represents the mean, error bars represent ±SEM.

Activity-dependent synaptic transmission within the intrahippocampal circuit facilitates adaptive behaviors for encoding spatial, episodic, and contextual memories (47–49). Building on our findings from single-nuclei multiomics that crotonate impacts chromatin accessibility and transcription of genes related to synaptic function (Fig. 2 *H* and *I* and *SI Appendix*, Fig. S9), we next sought to define which cellular signaling pathways are associated with these gene targets. Utilizing published databases of ligand–receptor interactions (50), we constructed an intercellular communication network of the principal neuronal subtypes to study the impact of Kcr enhancement on intrahippocampal signaling. Our analysis revealed that crotonate administration primarily increases the strength of glutamatergic signaling between the principal neuronal layers within the hippocampal circuit (Fig. 3*A*). Next, we investigated the ligand–receptor pairs in each intrahippocampal connection that exhibit increase in their communication probability following crotonate administration. We found that increasing Kcr levels strengthened the communication between distinct glutamatergic ligand–receptor pairs within specific neuronal networks of the hippocampal circuit. (Fig. 3*B*). These include the Slc1a2-Grik2/Grik4/Grik5, and Slc1a2-Gria4 pairs in the DG-CA3, Slc1a2-Grik2/Grik5/Grm7, and Slc1a1-Grik2/Grik5/Grm7 pairs in the CA3-CA1, and Slc1a1-Grik2/Grik5/Grm7, Slc1a2-Gria3/Gria4/Grik2/Grik5, and Slc17a7-Grik2/Gria3/Grm7/Grm8 pairs in the CA1-subiculum neuronal connection (Fig. 3*B*). We computed the communication probability of ligand–receptor interactions for glutamate signaling genes within principal neuronal cell types, which further confirmed the enhanced strength of glutamatergic transmission after crotonate administration (Fig. 3*C*). Collectively, our findings suggest that crotonate augments the strength of glutamatergic neurotransmission within the hippocampal circuit. Interestingly, such induction in glutamatergic gene expression was not observed when crotonate was administered under basal conditions without subthreshold SOR training. We performed RNAscope to examine the expression of a subset of glutamatergic genes (*Gria4* and *Grik2*) which we found to be induced in hippocampal CA1 in response to crotonate administration following subthreshold SOR training. We found that the expression of these two genes does not change in hippocampal CA1 following crotonate administration alone (*SI Appendix*, Fig. S13 *A* and *B*). These findings suggest that crotonate itself does not trigger glutamatergic gene expression. Instead, the transcriptional response requires both increases in histone lysine crotonylation and learning-induced neuronal activity.

To further underscore the role of Kcr in regulating glutamatergic gene expression, we employed an alternative approach to enhance histone Kcr levels by knocking down CDYL. We bilaterally transduced the dorsal hippocampus of WT mice with an AAV that encodes an shRNA targeting the CDYL protein (AAV_9_-U6-shCDYL). We first validated the efficacy of the viral construct by western blot analysis of whole-cell extracts from the dorsal hippocampus and observed a significant reduction in CDYL protein levels (*SI Appendix*, Fig. S14 *A* and *B*). Next, using immunofluorescence, we confirmed that CDYL knockdown resulted in significantly elevated Kcr levels in the hippocampal CA1 and CA3 subregions (*SI Appendix*, Fig. S14 *C* and *D*). We then examined the expression of select glutamatergic signaling genes, *Gria4* and *Grik2*, following subthreshold SOR training. Both transcripts were identified in our transcriptomic analyses as being upregulated in hippocampal CA1 in response to crotonate administration following subthreshold SOR training. Consistent with these findings, AAV-shCDYL-injected mice exhibited a similar upregulation of *Gria4* and *Grik2* in hippocampal CA1 (*SI Appendix*, Fig. S14 *E* and *F*). Thus, AAV-shCDYL manipulation recapitulates the glutamatergic gene expression changes observed following crotonate administration, further supporting a role for Kcr in regulating glutamatergic gene expression.

### Real-Time Ex Vivo Fluorescent Imaging Reveals that Crotonate Enhances Activity-Dependent Glutamate Transients and Postsynaptic Circuit Responsiveness.

Our single-nuclei multiomics and cell–cell communication analyses indicated enhanced glutamatergic signaling—a functional readout of which is presynaptic glutamate release. To test this, we transduced WT mice with an AAV encoding the fluorescent glutamate sensor iGluSnFR (pAAV_9_-hSyn-iGluSnFR) in the dorsal hippocampus. Four weeks later, acute hippocampal slices were prepared and incubated in either artificial CSF (aCSF) or aCSF containing 5 mM crotonate (Fig. 3*D*). We applied three stimulation frequencies (10, 20, and 50 Hz) based on prior studies (51) and recorded stimulus-evoked glutamate transients in CA1 following stimulation of CA3–CA1 Schaffer collateral afferents in the stratum radiatum (Fig. 3 *E*–*H*). Crotonate-treated slices exhibited a significant increase in peak glutamate amplitude across all three stimulation frequencies (Fig. 3*G*), with a concomitant increase in area under the curve at 10 and 20 Hz stimulation (Fig. 3*H*). In contrast, glutamate clearance kinetics (T_fast_ and T_slow_; depicted in a representative trace illustrated in *SI Appendix*, Fig. S15) were unchanged across conditions (*SI Appendix*, Fig. S16 *A* and *B*), suggesting that there is no difference in glutamate clearance. Together, these results indicate that crotonate augments glutamatergic neurotransmission by facilitating activity-dependent presynaptic glutamate release.

**Fig. 4.**
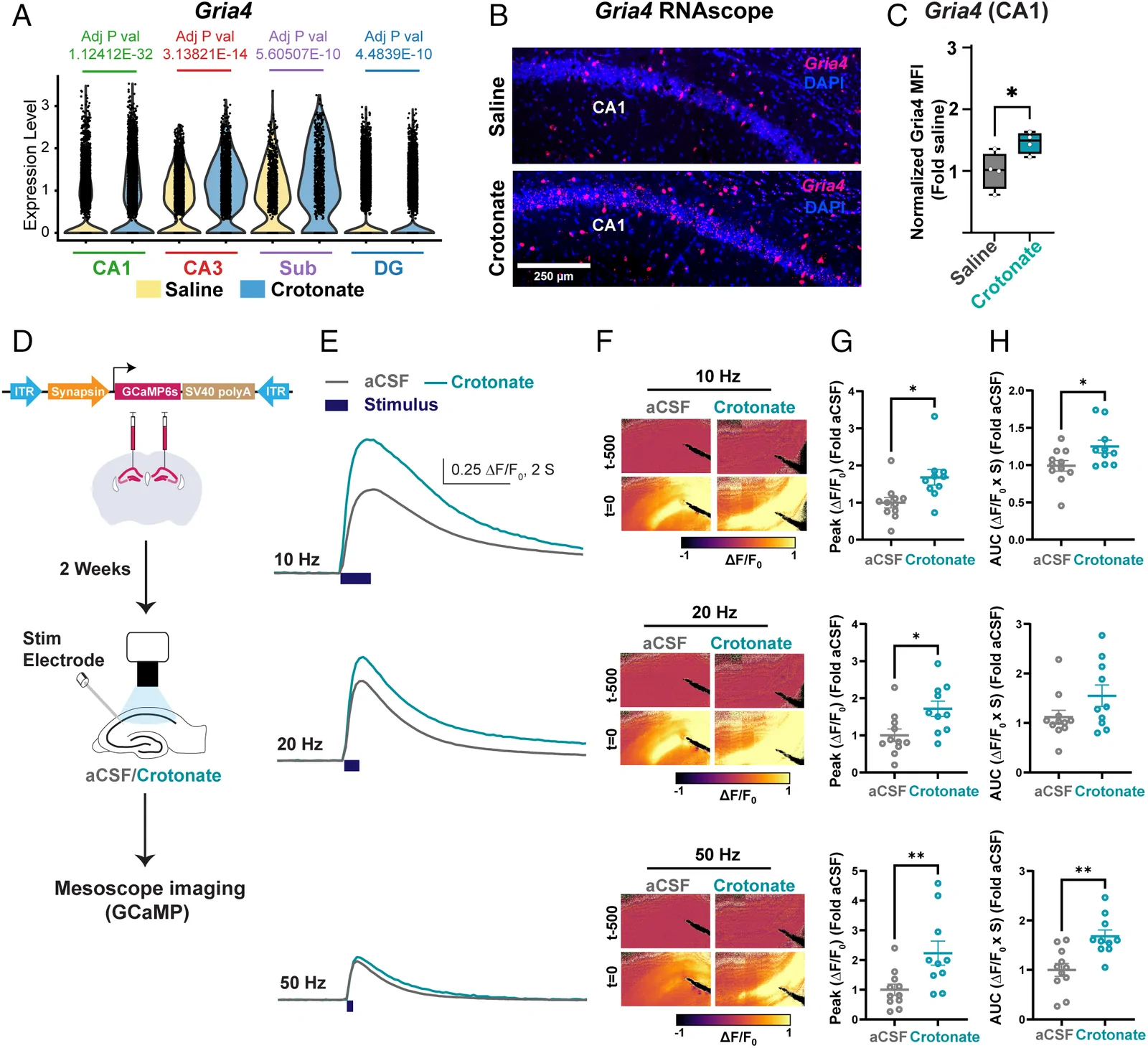
Kcr enhancement increases postsynaptic responsiveness at the circuit level. (*A*) Violin plot depicting expression of *Gria4* mRNA in hippocampal principal neuronal cell types across saline- and crotonate-treated conditions. (*B*) RNAscope validation of crotonate-mediated increases in *Gria4* expression in hippocampal CA1. Mice were trained with a suboptimal SOR training protocol, administered saline or crotonate (200 mg/kg) immediately after training, and perfused 1 h later. (Scale bar, 100 µm.) (*C*) Quantification of *B*. For CA1: Normalized Mean Fluorescent Intensity (MFI) of *Gria4* levels across the groups. Unpaired *t* test: t (6) = 2.613, *P = 0.04. Saline (n = 4) and Crotonate (n = 4), males only. (*D*–*H*) Kcr enhancement leads to larger stimulus-evoked calcium transients in acute hippocampal slices. (*D*) Schematic of the experimental design. (*E*) Representative traces of evoked calcium transient responses at 10, 20, and 50 Hz frequencies in control and crotonate (5 mM)-incubated slices. (*F*) Image panels showing stimulus-evoked calcium transients over time in the hippocampus upon stimulating the Schaffer collaterals in the CA1 Stratum Radiatum. The black object in each image depicts the stimulating electrode. t = 0 denotes the time point corresponding to the peak amplitude; t − 500 represents the time point 500 ms prior to t = 0. (*G*) The peak amplitude of evoked calcium transients was significantly higher in crotonate-treated slices compared to control across all the stimulation frequencies. Normalized Peak Amplitude across the groups. For 10 Hz: Lognormal Welch’s *t* test: t (17.84) = 2.803, *P = 0.0118. aCSF (n = 11) and Crotonate (n = 10). For 20 Hz (*Middle*): Lognormal Welch’s *t* test: t (17.18) = 2.734, *P = 0.014. aCSF (n = 11) and Crotonate (n = 10). For 50 Hz (*Bottom*): Lognormal Welch’s *t* test: t (18.98) = 3.134, **P = 0.0055. aCSF (n = 11) and Crotonate (n = 10). (*H*) The area under the curve of evoked calcium transients was significantly higher in crotonate-treated slices compared to control in the 10 Hz and 50 Hz stimulation groups. For 10 Hz: Lognormal Welch’s *t* test: t (18.06) = 2.260, *P = 0.0364. aCSF (n = 11) and Crotonate (n = 10). For 20 Hz (*Middle*): Lognormal Welch’s *t* test: t (18.43) = 1.702, *P* = 0.1056. aCSF (n = 11) and Crotonate (n = 10). For 50 Hz (*Bottom*): Lognormal Welch’s *t* test: t (13.52) = 3.241, **P = 0.0062. aCSF (n = 11) and Crotonate (n = 10). All scatter plots: the center line represents the mean, error bars represent ±SEM. All box plots: the center line represents the median, the box edges represent the top and bottom quartiles (25th to 75th percentiles), and the minimum and maximum whiskers.

Our iGluSnFR findings demonstrating increased presynaptic glutamate release complements our cell–cell communication analyses, altogether indicating enhanced glutamatergic signaling strength in response to crotonate. This led us to hypothesize that crotonate-mediated enhancement of activity-dependent presynaptic glutamate release at CA3–CA1 synapses would alter the circuit responsiveness to increased glutamatergic input. Such changes in circuit activity could be mediated, in part, by postsynaptic glutamate receptor genes that regulate activity-dependent calcium influx—the expression of some of these genes were found upregulated following crotonate administration. As a proof of concept, we investigated the expression profile of the AMPA receptor encoding gene *Gria4* (52), one of the significantly upregulated glutamatergic signaling genes following crotonate treatment. Consistent with the single-nuclei transcriptomic data (Fig. 4*A*), RNAscope analysis revealed a significant increase in *Gria4* expression in hippocampal CA1 after crotonate administration (Fig. 4 *B* and *C*).

We then employed an ex vivo fluorescence imaging approach using the genetically encoded calcium indicator GCaMP6s to examine widefield CA1 circuit dynamics in response to crotonate-dependent increase in activity-dependent presynaptic glutamate release from CA3 afferents (Fig. 4 *D*–*H*). WT mice were transduced with an AAV encoding GCaMP6s (pAAV_9_-hSyn-GCaMP6s-P2A-mRuby3) in the dorsal hippocampus, and acute slices were prepared 2 wk later. Slices were incubated in either crotonate (5mM) or aCSF (Fig. 4*D*), and stimulus-evoked glutamate transients were recorded over time at three different frequencies in CA1 following stimulation of CA3–CA1 Schaffer collateral afferents in the stratum radiatum (Fig. 4 *E* and *F*). We found that crotonate-treated slices exhibited significantly higher peak calcium transients across all three stimulation frequencies (Fig. 4*G*), accompanied by increased AUC at 10 and 50 Hz (Fig. 4*H*). To confirm that these effects were driven by transcriptional regulation rather than nongenomic actions of the drug, we performed a control experiment in which slices were incubated with crotonate for only 10 min (*SI Appendix*, Fig. S17 *A*–*C*). This brief exposure failed to alter either peak amplitude or AUC (*SI Appendix*, Fig. S17 *B* and *C*). Collectively, these findings indicate that crotonate enhances activity-dependent calcium signaling in hippocampal neurons—an effect likely attributable to the transcriptional upregulation of postsynaptic calcium-permeable glutamate receptor genes.

## Discussion

The discovery of various non-acetyl histone acylations over the past decade has led to studies to determine whether these modifications play a unique role in the epigenetic regulation of gene expression. Histone Kac has long been regarded as a cornerstone of the epigenetic mechanisms underlying long-term memory consolidation (2, 4, 10, 53). In this study, we demonstrate the critical role of lysine crotonylation, a non-acetyl histone acylation associated with transcription activation (6, 12, 27), in regulating hippocampal long-term memory consolidation. Through pharmacological and genetic approaches, we demonstrate the bidirectional modulation of long-term memory by manipulating Kcr levels in the dorsal hippocampus of adult mice. Mechanistically, our findings reveal that memory enhancement observed upon pharmacologically increasing Kcr levels is mediated through the function of ACSS2 in excitatory neurons in the hippocampus. Single-nuclei transcriptomic and epigenomic analyses revealed that crotonate primarily regulates the chromatin accessibility and expression of genes linked to synaptic function and glutamatergic neurotransmission within principal neuronal populations of the dorsal hippocampus. We then performed cell–cell communication analyses which predicted that crotonate administration during the critical window of memory consolidation enhances the communication probability of glutamatergic neurotransmission within intrahippocampal circuits. We performed ex vivo real-time fluorescence-based imaging and found that Kcr indeed facilitates activity-dependent presynaptic glutamate release and enhances neuronal excitability. Altogether, our study identified a novel epigenetic mechanism critical for long-term memory consolidation and excitatory neurotransmission.

Histone acylation profiles are regulated not just by the enzymatic activities of their respective writers and erasers, but also through changes in the nuclear concentrations of their metabolic precursors (6, 12). We found that bidirectional manipulation of hippocampal crotonyl-CoA levels had contrasting effects on memory consolidation. Specifically, administering crotonate increased histone Kcr levels in the dorsal hippocampus and enhanced long-term memory. Conversely, overexpressing CDYL reduced histone Kcr levels and led to deficits in long-term memory. Together, these findings not just establish Kcr as a key regulator of long-term memory but also identify CDYL as a potential “memory suppressor” gene (13). Crotonyl-CoA levels are also regulated by the metabolic enzyme ACSS2, which synthesizes crotonyl-CoA from crotonate (12, 32). In our study, we found that crotonate fails to increase hippocampal histone Kcr levels or enhance long-term memory in the ACSS2 cKO mice, indicating that ACSS2 is essential for converting crotonate into crotonyl-CoA, and that ACSS2-mediated conversion of crotonate to crotonyl-CoA is a critical molecular underpinning of Kcr-dependent enhancements in hippocampal memory.

Considering the promiscuity of histone acylation writers and erasers, the availability of metabolic precursors becomes even more critical in defining the landscape of histone acylations. Emerging evidence indicates that different acyl-CoA species compete for binding to KATs, such that changes in the relative abundance of specific acyl-CoAs can alter the balance of histone acylation marks (6, 12). For instance, depletion of cytoplasmic and nuclear acetyl-CoA pools in metazoans by knockdown of the enzyme ATP citrate lyase (ACLY) led to an increase in p300-catalyzed histone Kcr (54). Thus, increasing the nuclear availability of less abundant acyl-CoA species enhances their access to shared acyltransferases, which in turn facilitates the catalysis of histone acylation marks. Canonical Kac writers such as p300, and readers such as the YEATS-domain proteins, catalyze and recognize Kcr more efficiently (27, 55), and Kcr itself serves as a potent activator of transcription (12). Together, these observations raise the possibility that metabolically steering cells toward crotonyl-CoA utilization may represent an effective strategy to regulate transcriptional events during memory consolidation. In line with this idea, our findings show that pharmacologically increasing crotonyl-CoA levels to favor histone Kcr indeed offers a tractable and physiologically efficient approach to enhance long-term memory—laying the foundation for leveraging crotonate as a “memory enhancer” drug. Interestingly, recent studies have also shown that enhancing histone acetylation through acetate supplementation or glycerol triacetate administration can enhance long-term memory in WT mice (56) or ameliorate memory impairments in mouse models of Alzheimer’s disease (57). Elucidating cell type–specific transcriptomic signatures and chromatin accessibility changes underlying these memory-enhancing effects represents an exciting frontier for advancing our molecular understanding of how distinct histone acylations regulate gene expression during memory consolidation.

A key advantage of our multiomics framework is that it enabled us to identify cell-type-specific signatures of TF motif enrichment on genes that exhibit differential chromatin accessibility on their promoters and are undergoing transcription in response to crotonate treatment. Notably, binding motifs of several members of the Krüppel-like family of TFs, such as KLF15 and ZNF148, were found enriched in hippocampal subregions CA1, CA3, and subiculum following Kcr enhancement. These TFs—integral in regulating diverse developmental and homeostatic functions—are increasingly being recognized for their role in long-term memory and plasticity. For instance, *Klf15*-null mice show marked deficits in hippocampal-dependent memory, including reduced contextual freezing after aversive learning (58). Likewise, human genetic studies have shown that heterozygous variant in ZNF148 gene causes intellectual disability, whereas de novo mutations in ZNF148 have been associated with autism spectrum disorder, and cognitive impairment (59). Beyond the Krüppel-like family, we also observed enrichment of MAZ binding across the gene bodies of Kcr-responsive hippocampal DEGs. MAZ is known to recognize a 27-bp GC-rich region within the *GluN1* promoter, and binding of MAZ to *GluN1* is essential for its transcriptional activation (60). Collectively, these motif enrichments point to a regulatory architecture in which Kcr-mediated chromatin remodeling facilitates selective TF engagement with the genome, thereby regulating the transcriptional states that facilitate memory enhancement.

A major strength of our study derives from our findings that elucidate the role of crotonate in regulating glutamatergic neurotransmission. Our single-nuclei multiomics findings revealed that crotonate administration alters chromatin accessibility and upregulates genes involved in glutamatergic neurotransmission within excitatory neuronal populations. Cell–cell communication analysis—a powerful computational tool to infer and predict intercellular communication strength between neuronal populations within defined circuits (61), then further revealed that crotonate augments communication probability of intrahippocampal glutamatergic neurotransmission. Specifically, the analysis identified strengthened glutamatergic ligand–receptor interactions following crotonate treatment, exemplified by an increased predicted communication probability between presynaptic vesicular glutamate transporters (VGLUTs) and postsynaptic kainate and AMPA receptors (AMPARs). Together, these findings are suggestive of improved dynamics and fidelity of intrahippocampal glutamatergic signaling. These predictions were corroborated by ex vivo real-time imaging, which demonstrated elevated activity-dependent presynaptic glutamate release in the CA3-CA1 Schaffer collaterals and enhanced stimulus-evoked calcium transients in CA1 following crotonate treatment. Importantly, studies examining histone PTMs on genes involved in glutamatergic neurotransmission are limited (62, 63), and our understanding of how epigenetic mechanisms regulating glutamatergic gene expression lead to functional changes in synaptic neurotransmission and neuronal excitability remains in its early stages. Our work offers a mechanistic blueprint for how histone PTMs influence glutamatergic neurotransmission and neuronal activity at the circuit level. More importantly, these findings provide the conceptual groundwork for future in vivo studies examining how crotonate-mediated changes in glutamate transient dynamics regulate hippocampal circuit activity during memory encoding, and for extending this framework to determine how other histone acylations, such as acetylation, influence glutamatergic neurotransmission.

In summary, our findings demonstrate the critical role of a non-acetyl histone acylation which has not previously been studied in the context of long-term memory. By demonstrating that histone Kcr lies at the molecular crossroads of synaptic neurotransmission and behavior, our study provides a mechanistic framework for how epigenetic mechanisms translate into functional outcomes—shedding light on the elusive “histone code” of memory consolidation. Given that recent studies have demonstrated the role of Kcr-mediated regulatory mechanisms in several neurological disorders (39, 64), the broader relevance of our work is that it lays the foundation to develop more effective therapeutic interventions to treat cognitive impairments associated with neurological, neurodevelopmental, and neuropsychiatric disorders.

## Materials and Methods

All procedures involving mice were conducted in accordance with the U.S. NIH guidelines for the care and use of laboratory animals and were approved by the Institutional Animal Care and Use Committee (IACUC) at the University of Iowa. Detailed descriptions of the materials, experimental procedures, bioinformatic and imaging analyses, and statistical methods are provided in *SI Appendix*. Briefly, we employed adult male C57BL/6J and used stereotaxic delivery of AAVs into the dorsal hippocampus to manipulate CDYL expression and to express genetically encoded glutamate (iGluSnFR) and calcium (GCaMP6s) sensors. We administered AAV into the dorsal hippocampus of ACSS2^f/f^ mice to silence ACSS2 levels. Sodium crotonate was administered through oral gavage in mice or applied to acute hippocampal slices ex vivo. Histone extraction, western blotting, immunofluorescence, confocal imaging, and RNAscope-based in situ hybridization were performed to assess histone modifications, protein expression, and glutamatergic gene expression. Hippocampus-dependent long-term memory was evaluated using SOR and CFC paradigms. Single-nuclei multiomic sequencing (10× Genomics) was used to examine cell-type-specific transcription and chromatin accessibility, followed by DEG, differential chromatin accessibility, gene ontology, transcription-factor motif enrichment, and cell–cell communication analyses. Acute hippocampal slices expressing iGluSnFR or GCaMP6s were used for mesoscopic imaging of stimulus-evoked glutamate and calcium transients following stimulation. Behavioral, biochemical, molecular, and imaging datasets were analyzed using appropriate parametric or nonparametric statistical tests and multiple-comparison procedures as described for each experiment.

## Supplementary Material

Appendix 01 (PDF)

Dataset S01 (XLSX)

Dataset S02 (XLSX)

Dataset S03 (XLSX)

Dataset S04 (XLSX)

Dataset S05 (XLSX)

## Data Availability

Single-nuclei multiomics (RNA-seq + ATAC-seq) data have been deposited in NCBI Gene Expression Omnibus (GEO) (GSE281007, https://www.ncbi.nlm.nih.gov/geo/query/acc.cgi?acc=GSE281007) (65). The code used for the analyses to generate the figures related to single-nuclei multiomics data can be accessed through GitHub (https://github.com/ChatterjeeEpigenetics/CrotonylationMultiomics2024) (66). All other data are included in the manuscript and/or supporting information.
