# Supplementary material for: Lysine crotonylation acts as an epigenetic switch for glutamate neurotransmission and spatial memory
Source: Proc Natl Acad Sci U S A. 2026 Oct 28;123(44):e2608460123. doi: 10.1073/pnas.2608460123 (PMC13732231; doi:10.1073/pnas.2608460123)
Supplement: Supplementary file 1 [file pnas.2608460123.sapp.pdf]

Supporting Appendix

Lysine Crotonylation Acts as an Epigenetic Switch for Glutamate Neurotransmission and Spatial Memory

Utsav Mukherjee[1,2,3], Budhaditya Basu[1,2], Pratyush Suryavanshi [2,7], Stacy E. Beyer[1,2], Saaman Ghodsi[1,2], Nathan Robillard[1,2], Yann Vanrobaeys[1,2,4], Eric B. Taylor[5,6], Joseph Glykys[2,7], Ted Abel[1,2*], and Snehajyoti Chatterjee[1,2*]

[1]Department of Neuroscience and Pharmacology, Carver College of Medicine, University of Iowa, Iowa City, IA 52242, United States

[2]Iowa Neuroscience Institute, University of Iowa, Iowa City, IA 52242, United States

[3]Interdisciplinary Graduate Program in Neuroscience, University of Iowa, Iowa City, IA 52242, United States

[4]Interdisciplinary Graduate Program in Genetics, University of Iowa, Iowa City, IA 52242, United States

[5]Fraternal Order of Eagles Diabetes Research Center, University of Iowa, Iowa City, IA 52242, United States

[6]Department of Molecular Physiology and Biophysics, Carver College of Medicine, University of Iowa, Iowa City, IA, 52242, United States

[7]Stead Family Department of Pediatrics, Carver College of Medicine, University of Iowa, Iowa City, IA, 52242, United States

[*]Corresponding authors: ted-abel@uiowa.edu and snehajyoti-chatterjee@uiowa.edu

This file includes Supplementary Methods, Supplementary Figures (1-17), Supplementary Figure Legends (1-17), and Supplementary Table Legends (1-5).

Supplementary Methods

Data reporting: No statistical methods were applied to predetermine sample size.

***Mouse lines*:** Adult male C57BL/6J mice were purchased from Jackson Laboratories. The ACSS2$^{f/f}$ mice were generated at the University of Iowa Genome Editing Facility(1). This mouse model was created by inserting loxP sites flanking the Exon2 of ACSS2 using CRISPR/Cas9. The mice were 2-4 months of age at the time all the behavioral and biochemical experiments were performed. All mice had ad libitum access to food and water, and lights were maintained on a 12-hr light/dark cycle. All experiments were conducted according to US National Institutes of Health guidelines for animal care and use and approved by the Institutional Animal Care and Use Committee of the University of Iowa, Iowa.

***Adeno-associated virus constructs and stereotactic surgeries*:** AAV_9-CaMKIIα-EGFP, AAV_9-CaMKIIα-CDYL-V5, AAV_9-U6-shCDYL, and AAV_9-U6-scambled were purchased from VectorBuilder (VectorBuilder Inc). AAV_9-CaMKIIα-GFP-Cre (#105551), $pAAV_9$-hSyn-iGluSnFr (#98929), and $pAAV_9$-hSyn1-GCaMP6s-P2A-mRuby3 (#112005) were purchased from Addgene. Mice were anesthetized using 5% isoflurane. A steady flow of 2.5% isoflurane was maintained throughout the remainder of the stereotactic surgery. 1 µl of respective AAVs were bilaterally injected into the dorsal hippocampus (coordinates: anteroposterior, −1.9 mm, mediolateral, ±1.5 mm, and 1.5 mm below bregma). Following viral infusion, drill holes were closed with bone wax (Lukens) and the incisions were sutured. Intraperitoneal (IP) injections of Meloxicam (5 mg/kg) were administered for 2 successive days after the surgery to manage the post-operative pain.

Drug preparation: Sodium crotonate was freshly prepared on the day of the experiment. 2.5 gm of Crotonic acid (Sigma) was dissolved in 30 ml of 0.9% saline. Thereafter, the pH of the solution was adjusted to 7.4 using 5(N) NaOH, and additional 0.9% saline was added to adjust the final volume to 50 ml. The solution was then filtered through a 0.2 µm syringe filter. Appropriate volume of the drug (calculated based on the body weight of the animals) was administered via oral gavage based on the dosing regimen.

Histone extraction: Histone extraction was performed using a commercially available kit (Histone Extraction Kit, Active Motif, #40028) according to the manufacturer's protocol. Briefly, flash frozen hippocampi were mechanically homogenized in 300 µl of ice-cold Lysis Buffer AM8 using Dounce homogenizers and incubated on ice for 30 mins. The homogenate was centrifuged at 2,644 x g for 2 minutes at 4°C, and the nuclear pellet was resuspended in 250 µl ice-cold

Extraction buffer. Following resuspension, the nuclear suspension was incubated on an end-to-end rotator overnight at 4°C. The pellet insoluble material was centrifuged at 20,800 x g for 10 minutes at 4°C the next day, the supernatant was collected, and the requisite volume of Complete Neutralization Buffer was added. Samples were stored in -80°C prior to western blotting.

Western blot analysis: Western blotting was performed as previously described(2, 3). Whole cell lysates were run on a 4–20% Tris-HCl Protein Gel (BIO-RAD, #3450033). Proteins from the gel were then transferred to methanol-activated polyvinylidene difluoride membranes using the Trans-Blot Turbo Transfer System (BIO-RAD). Membranes were blocked with the Odyssey® Blocking Buffer (LI-COR) diluted in TBS and incubated overnight at 4°C with the following primary antibodies: pan-Kcr (1:1000, PTM BIO, #PTM-501), Histone H3ac (pan-acetyl) (1:1000, ACTIVE MOTIF, # 39139), Total H3 (1:5000, ACTIVE MOTIF, #61647), ACSS2 (1:1000, Cell Signaling, #3658), CDYL (1:1000, Sigma # SAB2103346) and Actin (1:10,000, ThermoFisher Scientific, #MA1-91399). Post primary antibody incubation, membranes were washed thrice in TBS for 5 mins and incubated with IRDye® 800CW Goat anti-Rabbit IgG Secondary Antibody (1:5,000, LI-COR, #926-32211) and IRDye® 680LT Goat anti-Mouse IgG Secondary Antibody (1:5000, LI-COR, #926-68020). Membranes were then washed thrice in TBS, 5 mins each. Images were acquired using the Odyssey Infrared Imaging System (LI-COR). Quantification of western blot bands was performed using Image Studio Lite ver5.2 (LI-COR).

Immunofluorescence (IF)**:** IF experiments were performed as per earlier studies(2, 3). Animals were perfused with 4% PFA. Whole brains were harvested, immersed in 4% PFA, and stored at 4°C. 24 hrs after, brains were immersed in 30% sucrose and stored at 4°C. 20 µm coronal brain sections were made in a cryostat (Leica). Free-floating sections were washed thrice with PBS, blocked in a blocking buffer (0.1% PBST and 5% BSA) and incubated with the following primary antibodies for 48 hr: pan-Kcr (1:2000, PTM BIO, #PTM-501), CDYL (1:500, Sigma, #SAB2103346), V5 (1:500, ThermoFisher Scientific, #37-7500), and GFP (1:4000, Abcam, #ab290). Post primary antibody incubation, sections were washed in PBS thrice, followed by a 2 hr secondary antibody incubation with the following antibodies: Goat anti-Mouse IgG (H+L) Cross-Adsorbed Secondary Antibody, Alexa Fluor™ 546 (1:500, ThermoFisher Scientific, #A-11003), Goat anti-Rabbit IgG (H+L) Cross-Adsorbed Secondary Antibody, Alexa Fluor™ 647 (1:500, ThermoFisher Scientific, #A-21244). Sections were then washed thrice in PBS and mounted on Superfrost™ Plus microscope slides (Fisherbrand). This was followed by coverslip mounting with ProLong™ Diamond Antifade Mountant with DAPI (ThermoFisher Scientific, #P36962).

Confocal imaging and image analysis: Confocal images of IHC experiments were obtained in an Olympus FV 3000 confocal microscope using a 20X NA = 0.4 achromat dry objective at 800 × 800-pixel resolution and 1X optical zoom. All images (16 bit) were acquired with identical settings for laser power, detector gain and pinhole diameter for each experiment and between experiments. Images from the different channels were stacked and projected at maximum intensity using ImageJ (NIH). Mean fluorescence intensity (MFI) was computed using ImageJ plugins. MFIs were then subtracted to background intensities in each slice. Background subtracted MFIs in the experimental group were then normalized to the average background subtracted MFIs in control. For MFI quantification, the entire area corresponding to CA1, CA3, DG or subiculum were considered as region of interest.

In situ hybridization (RNAscope): *In situ* hybridization was performed using the RNAscope™ Multiplex Fluorescent Reagent Kit v2 (Advanced Cell Diagnostics) according to the manufacturer's protocol as previously described(3). Briefly, 20 μm cryosections obtained from fixed frozen brains were mounted on Superfrost™ Plus microscope slides (Fisherbrand). Slides then underwent serial dehydration in Ethanol, followed by Hydrogen Peroxide treatment, Target Retrieval, and Protease III treatment. Hybridization of probes was done at 40°C for 2 hr in a HybEZ oven using probes against *Gria4* and *Grik2.* The probe signal was amplified with Pre-amplifiers (AMP 1-FL, AMP 2-FL, and AMP 3-FL) and counterstained with OPAL 520 and OPAL 570 reagents (FP1488001KT, Akoya Biosciences). Finally, coverslip mounting was done on the slides using ProLong™ Diamond Antifade Mountant with DAPI (ThermoFisher Scientific, P36962). The slides were stored in 4^0C until they were imaged.

Spatial object recognition (SOR) task**:** All the behavioral experiments were performed based on previously published studies(2, 3) during the light cycle in between Zeitgeber time (ZT) 0 through 2. Mice were individually housed for 7 days before the training. Animals were handled for 2 mins each day for 5 successive days before training. In the strong training paradigm, animals were habituated in an open field for 6 minutes. This was followed by three 6-minute sessions inside the same arena containing three different glass objects. These objects were placed at specific spatial coordinates with respective to a spatial cue within the arena. An inter-trial interval of 5 minutes was set in-between the three training sessions. In the sub-threshold training paradigm, mice were subjected to three 3-minute training trials in the open field with two objects and an inter-trial interval of 5 minutes in between sessions. 24 hr after training, the animals were returned to the arena with one of the objects displaced to a novel spatial coordinate. Exploration

time around all the objects were then manually scored. Percent preference towards the displaced object was calculated using the following equation:

$$\textit{Percent preference for displaced object} = \frac{(exploration\ towards\ the\ displaced\ object)}{(total\ exploration\ towards\ all\ objects)}\ x\ 100$$

For the histone Kcr profiling and the single-nuclei experiments, animals were euthanized by cervical dislocation 1 hr after the last training trial in the SOR task. Hippocampal tissue was flash frozen and stored at -80^{0}C. Home caged animals were euthanized within the same ZT window to eliminate the possible confounding effects of circadian rhythmicity. To examine the learning-induced expression of Kcr in hippocampal subregions, mice were perfused 1 hr after the training session, and whole brains were harvested for IF.

Contextual fear conditioning: Contextual fear conditioning was performed using a sub-threshold learning paradigm. In brief, mice were handled daily for five days before conditioning. The conditioning protocol involved a single 2-second, 0.75 mA footshock delivered 2.5 minutes after the mice were introduced into the chamber. Mice remained in the chamber for an additional 30 seconds before being returned to their home cage. 24-hrs later, they were reintroduced to the same chamber for 5 mins. Freezing behavior was assessed using FreezeScan software (CleverSys Inc.).

Nuclei isolation: Nuclei isolation from frozen hippocampal tissue was performed according to the manufacturer's protocol (Chromium Nuclei Isolation Kit with RNAse Inhibitor, 10x Genomics, #1000494). Briefly, frozen tissue was homogenized in 500 µl of Lysis Buffer using Dounce homogenizers. The lysate was transferred to a Nuclei Isolation Column and centrifuged at 16,000 x g for 30 seconds at 4°C. The pellet was then resuspended in 500 µl of Debris Removal Buffer and centrifuged at 700 x g for 10 minutes at 4°C. The nuclear pellet was then resuspended with 1 ml of Wash Buffer and centrifuged at 500 x g for 5 minutes at 4°C. Finally, the nuclear pellet was resuspended in 50 µl of Resuspension Buffer. Nuclei count was manually done using a Hemocytometer.

Single-nuclei multiomic data processing and analysis: Raw sequencing data were processed using the 'Cell Ranger ARC' pipeline (v2.0.2) with the cell ranger-arc mm10 reference. Default parameters were used to align reads, count unique fragments or transcripts, and filter high-quality nuclei. HDF5 files for each sample (Saline1, Saline2, Crotonate1, Crotonate2) containing barcoded RNA counts and ATAC fragments per cell cluster were loaded into Seurat (Read10X_h5). This resulted in the generation of four Seurat objects, each containing both RNA

and ATAC assays. Nuclei with outliers within the ATAC and RNA QC metrics (<200 and >100,000 ATAC read counts, <200 and >50,000 RNA read counts, nucleosomal signal > 4, TSS enrichment < 3, %reads in peaks < 15 and percentage of mitochondrial reads > 5) were removed.

To analyze the RNA component of the multiomics data, gene counts were normalized, and log transformed (LogNormalize). The top 2,000 most variable features that distinguish each cell were identified using 'FindVariableFeatures' (selection.method = 'vst'). Features that are repeatedly variable across cells and datasets were selected for integration ('SelectIntegrationFeatures'). We then identified anchors ('FindIntegrationAnchors''), which took the list of 4 individual Seurat objects for each sample as input. These anchors were used to integrate the four datasets together ('IntegrateData'). Linear dimensionality reduction was performed on the integrated Seurat object by principal component analysis (runPCA, npcs = 30). A k-nearest-neighbours graph was constructed based on Euclidean distance in PCA space and refined ('FindNeighbors'), following which the nuclei were clustered using the Louvain algorithm (FindClusters, resolution = 0.5). Clusters were visualized with UMAP (runUMAP, dims = 30). Both RNA and ATAC assays were used to identify cell-type specific signatures of biomarkers. Differentially expressed genes (DEGs) in individual clusters between saline and crotonate treated groups were calculated (FindMarkers, test.use = 'wilcox,' Padj < 0.05, absolute logFC.threshold of 0.2).

To analyze the ATAC component of the multiomics data, the default assay was switched to ATAC prior to integrating the four Seurat objects, and peak calling was performed. The set of peaks identified by 'Cellranger' often merges distinct peaks that are close together - confounding the motif enrichment analysis and peak-to-gene linkage. We were able to circumvent this concern and identify a more accurate set of peaks by using the 'MACS2' (CallPeaks) peak calling feature on all cells together. Peaks on nonstandard chromosomes and in genomic blacklist regions were removed ('keepStandardChromosomes' and 'subsetByOverlaps'). A frequency-inverse document frequency normalization was performed across cells and peaks ('RunTFIDF'). Thereafter, a feature selection was performed using all the peaks as input (FindTopFeatures, min.cutoff = 5). The selected peaks went through a dimensional reduction on the TF-IDF normalized matrix using a singular value decomposition ('RunSVD'). To identify the differentially accessible regions (DAR) between crotonate versus saline group, the Seurat function 'FindMarkers' was used using logistic regression (LR) as a method to test significance. The DARs with adjusted p value < 0.05 and absolute log2foldchange threshold of above 0.2 were considered as significantly differentially accessible. Further, the DARs were annotated using 'Closestfeature' function from Signac package as well as 'annotatePeak' function from 'ChIPseeker' package. The DEGs were furthered

correlated with DARs where the DARs were selected only from the promoter (+/- 2kb from transcription start site) and genebody regions, filtering out the peaks from distal genomic regions and downstream of 3'UTR(4). The genes found concordant in both DEG and DAR lists were shortlisted for downstream gene ontology enrichment analysis. UpSet plots were generated using UpSetR package.

Gene Ontology enrichment analysis: The concordant DARs and DEGs were analyzed for Molecular Function enrichment analysis by using the 'clusterProfiler' package with the default criteria (pvalueCutoff = 0.01 and qvalueCutoff = 0.05). Here, the significant upregulated DEGs (adj p value < 0.05 and log2FC > 0.2) concordant with the significant more accessible DARs (adj p value < 0.05 and log2FC > 0.2) were used to generate the upregulated gene list, whereas the significant downregulated DEGs (adj p value < 0.05 and log2FC < -0.2) concordant with the significant less accessible DARs (adj p value < 0.05 and log2FC < -0.2) were used to generate the downregulated gene list. The DARs were selected only from the promoter (+/- 2kb from transcription start site) and gene body regions, filtering out the peaks from distal genomic regions and regions downstream of 3'UTR. Data visualizations were made using clusterProfiler package. Ridgeline charts were generated using free online tool ExpressAnalyst (https://www.expressanalyst.ca/ExpressAnalyst/ModuleView.xhtml) using the concordant DARs and DEGs.

TF motif enrichment analysis: To identify cell-type specific regulatory sequences, we performed transcription factor motif enrichment analysis on the DEGs that were found to have concordant differentially accessible peaks in the promoter region. Here, we restricted TF motif enrichment only in the promoter region (a window of 2000bp upstream and downstream of transcription start site). Motif enrichment was performed using 'FindMotifs' function of signac package. The motifs that had adjusted p value < 0.05 were considered significant. The top 15 significant motifs from the clusters were plotted as heatmap using ComplexHeatmap package. Weight matrices for the top motifs were also plotted to visualize the motif sequences.

Cell-cell communication analysis: The cell-cell communication analysis on the snRNA-seq data was performed using CellChat (v2.1.2). The Saline and Crotonate RNA normalized counts were taken and individual cellchat objects were created. Ligand-receptor (LR) interactions in each group were calculated by identifying overexpressed ligands or receptors with a log fold change cutoff 0.1 (identifyOverExpressedGenes(thresh.fc = 0.1, thresh.p = 0.05)) followed by identifying interactions if LR pairs are overexpressed (identifyOverExpressedInteractions). To assign each

interaction with a probability score, computeCommunProb function was used with a default statistical method called 'trimean'. Cell-cell communication was filtered to have minimum cell number 10 in each cell group (filterCommunication(min.cells = 10)). After that communication probability on signaling pathway level was calculated by summarizing the communication probabilities of all LR interactions associated with each signaling pathway. Finally, the cell-cell communication network was aggregated by counting the number of links or summarizing the communication probability amongst the cell groups. Thereafter, the cellchat objects were merged and compared using netVisual_aggregate, netAnalysis_signalingChanges_scatter, and netVisual_chord_gene functions.

Acute brain slice preparation: Acute hippocampal slices were prepared as described in published studies(5). 2-4-month-old male mice were deeply anaesthetized with isoflurane, following which their brains were harvested for slice preparation. Coronal sections were obtained in ice-cold dissection buffer (in mM: 220 sucrose, 3.3 KCl, 5 $MgSO_4$, 1.25 NaH_2PO4, 25 $NaHCO_3$, 11 D-glucose, and 0.2 $CaCl_2$) and sections containing dorsal hippocampal representations were allowed to recover in a chamber containing normal artificial CSF (aCSF) (in mM; 116 NaCl, 3.3 KCl, 1.3 $MgSO_4$, 1.25 NaH_2PO4, 25 $NaHCO_3$, 11 D-glucose, 1.3 $CaCl_2$) saturated with carbogen (95% O_2 / 5% CO_2) at 32°C. The slices were incubated for 2 hrs either in normal aCSF or 5 mM Crotonate before being transferred to a submerged chamber for imaging, constantly supplied with aCSF (flow rate: 2.5 ml/min) also maintained at 32°C. For the experiment described in **Fig. S17**, the slices were incubated for 5-10 mins with the drug, following which they were transferred to the submerged chamber containing aCSF for imaging.

Mesoscopic imaging and image analysis: Acute coronal sections containing hippocampi transduced with either iGluSnFr or GCaMP6s were placed in a submerged chamber attached to the stage of an upright microscope (Olympus MVX10), continuously perfused with aCSF saturated with carbogen and maintained at 32-34°C. A monopolar stainless steel stimulation electrode (AM systems) was placed on in the Stratum Radiatum to stimulate the Schaffer Collateral pathway. Optical excitation was delivered by an LED light (X-Cite XYLIS XT720S). Fluorescent mesoscopic imaging of the entire brain slice was performed using a 2x objective (NA 0.5). The LED light was band-passed through a 470/40 nm filter and a dichroic 495 nm filter, while the emitted light was isolated using a 525/50 nm filter. For stimulus trains, 10 stimuli (3mV, 500 µs) were delivered with variable inter-stimulus intervals (for 10 Hz: 100ms; for 20Hz: 50ms; for 50Hz: 20ms), as done in previous studies(5). Images were acquired with a CMOS camera (Basler Ace acA4112-30µm) at a frame rate of 10 Hz (GCaMP6s imaging) or 90-100 Hz (iGluSnFr

imaging) with 4:1 binning. The camera acquisition and LED excitation were digitally synchronized using Micro-manager (ImageJ). Raw images in time series were background-subtracted and smoothed (median filter). Time series were baseline subtracted, scaled to baseline means ($\Delta F/F_0$, baseline: ~2s before the stimulation / response onset), and then compressed into Max Intensity Projections (MIPs). For Glutamate transients (iGluSnFr), responsive regions (ROIs) were generated by converting the $\Delta F/F_0$ MIPs into binary (Threshold: mean + SD). For calcium transients, hippocampal area that showed ectopic expression of GCaMP6s was selected as the ROI. Fluorescence traces generated using the respective ROIs were analyzed in MATLAB (MathWorks). Ca^{2+}/ Glutamate response parameters (peak amplitude, locations, width, prominence) were determined using the 'FindPeaks' function in MATLAB (minimum peak prominence: 0.025 for Glutamate, 0.05 for Ca^{2+}). Area under the curve (1) was then calculated using trapezoidal numerical integration ('Trapz' function) in MATLAB. For glutamate transients, the slow and fast components of the decay kinetics (Tau) were determined by fitting two single-term exponential fits to the decay of the response (τ_{fast}: stimulus offset to ~4s, τ_{slow}: ~4s to 10s).

Statistics: Behavioral and biochemical data were analyzed using unpaired two-tailed t-tests, one-way ANOVA, or two-way ANOVAs (sometimes with repeated measures as the within-subject variable). Sidak's multiple comparison tests or Dunnett's multiple comparison tests were used for post-hoc analyses wherever required. In the iGluSnFR and GCaMP6s imaging data, normality of the distributions was assessed using the D'Agostino–Pearson K2, Anderson-Darling tests, and QQ plots. If data was log-normal, a Lognormal Welch's t test was used. Outliers were detected using Grub's or IQR tests (for parametric and nonparametric data), although no outliers were detected or excluded. In all the experiments, differences were considered statistically significant when $p<0.05$. As indicated for each figure panel, all data were plotted in box plots.

References

1. A. J. Rauckhorst *et al.*, A hierarchical hepatic de novo lipogenesis substrate supply network utilizing pyruvate, acetate, and ketones. *Cell Metab* 10.1016/j.cmet.2024.10.013 (2024).
2. S. Chatterjee *et al.*, Endoplasmic reticulum chaperone genes encode effectors of long-term memory. *Sci Adv* **8**, eabm6063 (2022).
3. Y. Vanrobaeys *et al.*, Mapping the spatial transcriptomic signature of the hippocampus during memory consolidation. *Nat Commun* **14**, 6100 (2023).
4. T. Stuart, A. Srivastava, S. Madad, C. A. Lareau, R. Satija, Single-cell chromatin state analysis with Signac. *Nat Methods* **18**, 1333-1341 (2021).
5. P. Suryavanshi, P. Sawant-Pokam, S. Clair, K. C. Brennan, Increased presynaptic excitability in a migraine with aura mutation. *Brain* **147**, 680-697 (2024).

Supplementary Figures

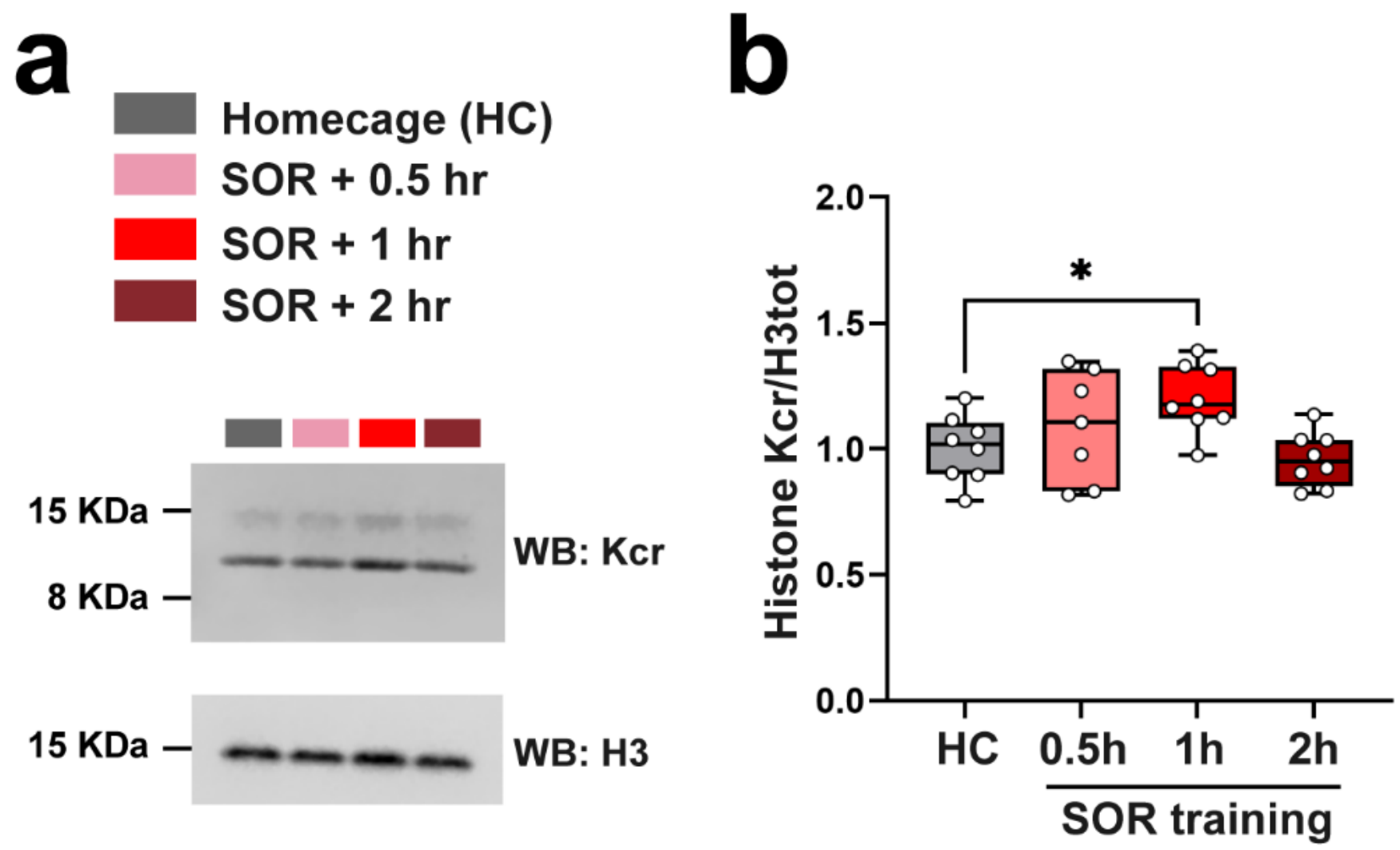

Supplementary Figure S1. Learning-dependent increase in histone lysine crotonylation in the dorsal hippocampus. a. Western blot showing histone crotonylation analyzed from the dorsal hippocampus of mice trained in spatial object recognition (SOR) task and euthanized at 0.5, 1, or 2h after training. Homecage (6) mice were used as controls. **b.** Quantification of **a.** One-way ANOVA: Kcr: $F(3, 27)=3.953$, $p= 0.0185$. Dunnett multiple comparisons tests: *$p=0.0379$ (HC versus 1 hr). Homecage (n = 8), SOR + 0.5 hr (n = 7), SOR + 1 hr (n=8), SOR + 2 hr (n=8), males only. All box plots: the center line represents the median, the box edges represent the top and bottom quartiles (25th to 75th percentiles), and the minimum and maximum whiskers.

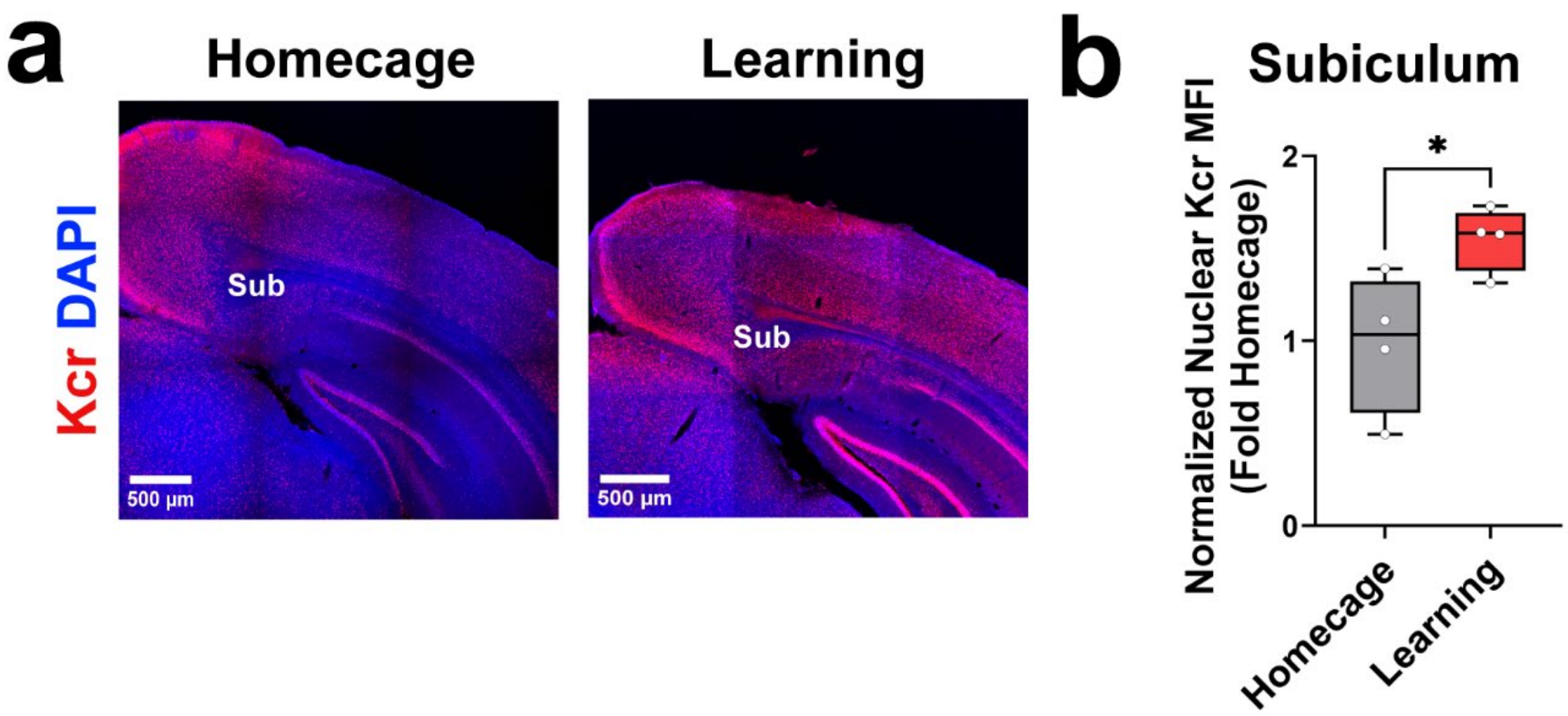

Supplementary Figure S2. Spatial learning elevates lysine crotonylation levels in the Subiculum. a. Immunofluorescence using anti-Kcr antibody showing levels of Kcr in Subiculum (Sub) of HC and learning (SOR+1hr) mice. **b.** Quantification of **a.** Unpaired t test: $t(6) = 2.733$, $^{*}p = 0.034$. Homecage ($n = 4$) and SOR+1 hr ($n = 4$), males only. All box plots: the center line represents the median, the box edges represent the top and bottom quartiles (25th to 75th percentiles), and the minimum and maximum whiskers.

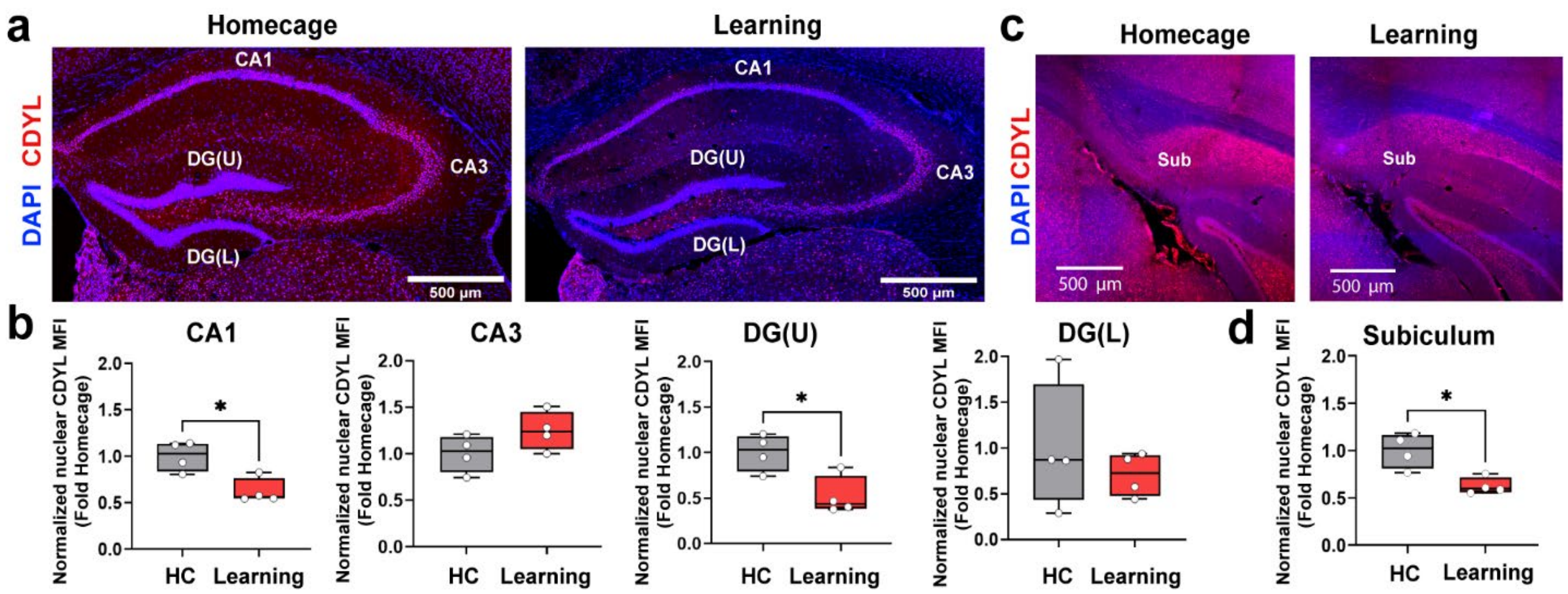

Supplementary Figure S3. Spatial learning reduces hippocampal CDYL levels. a-d. Immunofluorescence using an anti-CDYL antibody was performed on brain slices from mice 1 hr after training in the SOR task. Hippocampal sub-regions CA1 (**3a**), CA3 (**3a**), DG upper blade (U) (**3a**), DG lower blade (L) (**3a**), and Subiculum (**3c**) were examined. Normalized Mean Fluorescent Intensity (MFI) of nuclear CDYL levels across the groups. **b, d.** Quantification of **a** and **c** respectively. For CA1 (**3b**): Unpaired t test: t (6) = 3.598, *p = 0.0114. Homecage (n = 4) and SOR+1 hr (n = 4), males only. For CA3 (**3b**): Unpaired t test: t (6) = 1.704, p = 0.1393. Homecage (n = 4) and SOR+1 hr (n = 4), males only. For DG upper blade (**3b**): Unpaired t test: t (6) = 3.254, *p = 0.0174. Homecage (n = 4) and SOR+1 hr (n = 4), males only. For DG lower blade (**3b**): Unpaired t test: t (6) = 0.7837, p = 0.463. Homecage (n = 4) and SOR+1 hr (n = 4), males only. For Subiculum (**3d**): Unpaired t test: t (6) = 3.657, *p = 0.0106. Homecage (n = 4) and SOR+1 hr (n = 4), males only. All box plots: the center line represents the median, the box edges represent the top and bottom quartiles (25th to 75th percentiles), and the minimum and maximum whiskers.

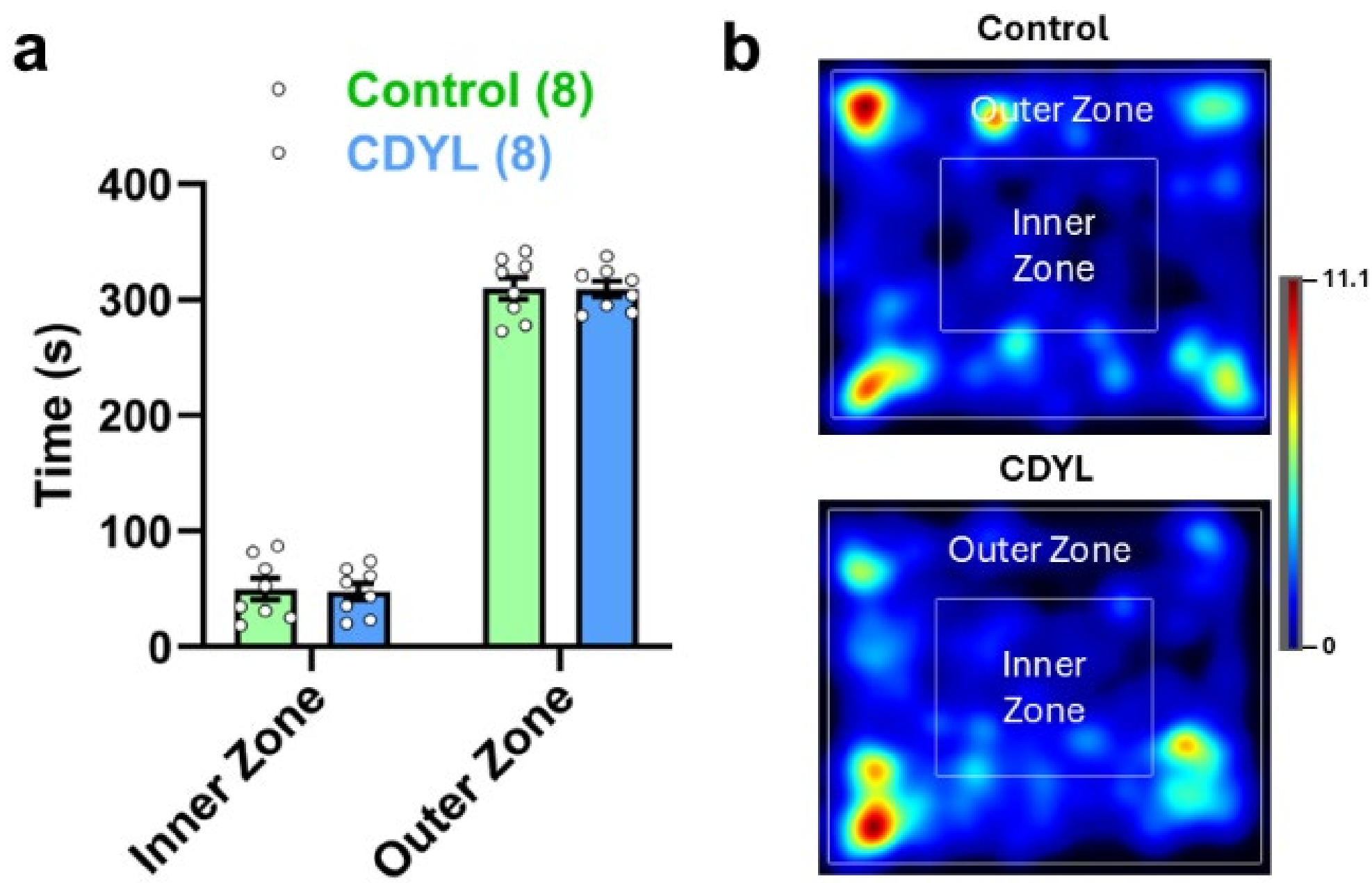

Supplementary Figure S4. Overexpression of CDYL does not exert any anxiety like behavior in mice. a. Quantification of the time spent in the Inner and Outer Zones of the open arena during habituation**. b.** Representative heat maps showing the time spent in Inner and Outer Zones.

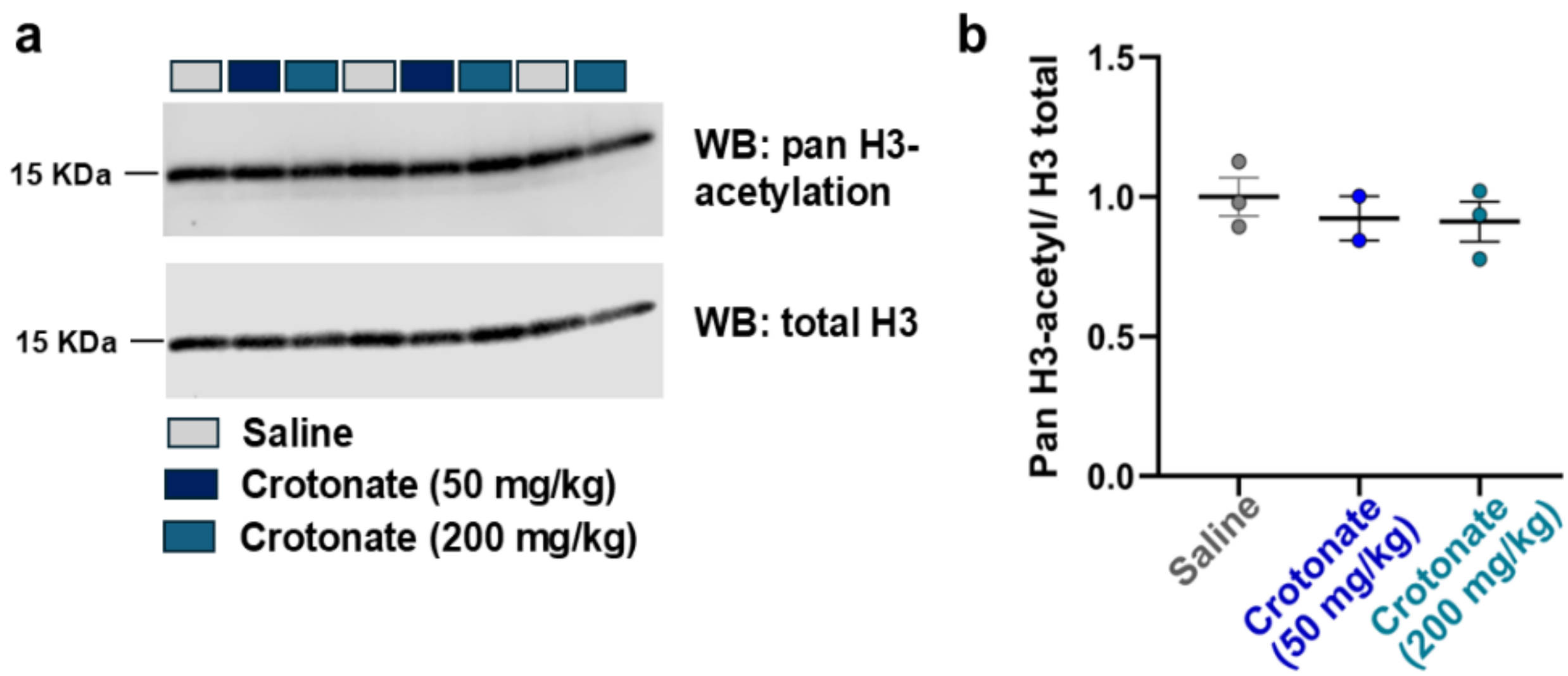

Supplementary Figure S5. Crotonate gavage does not alter histone acetylation levels in the dorsal hippocampus. a. Western blot images depicting pan H3-acetylation and their corresponding total H3 levels across groups. **b.** Quantification of **a.** One-way ANOVA: pan H3-acetyl: $F (2, 5) = 0.474$, $p = 0.6479$. Dunnett multiple comparisons tests: $p = 0.7204$ (Crotonate 50 mg/kg vs Saline); $p = 0.6015$ (Crotonate 200 mg/kg vs Saline). Saline (n = 3), Crotonate 50 mg/kg (n=2), Crotonate 200 mg/kg (n=3); males only. Error bars represent ± SEM.

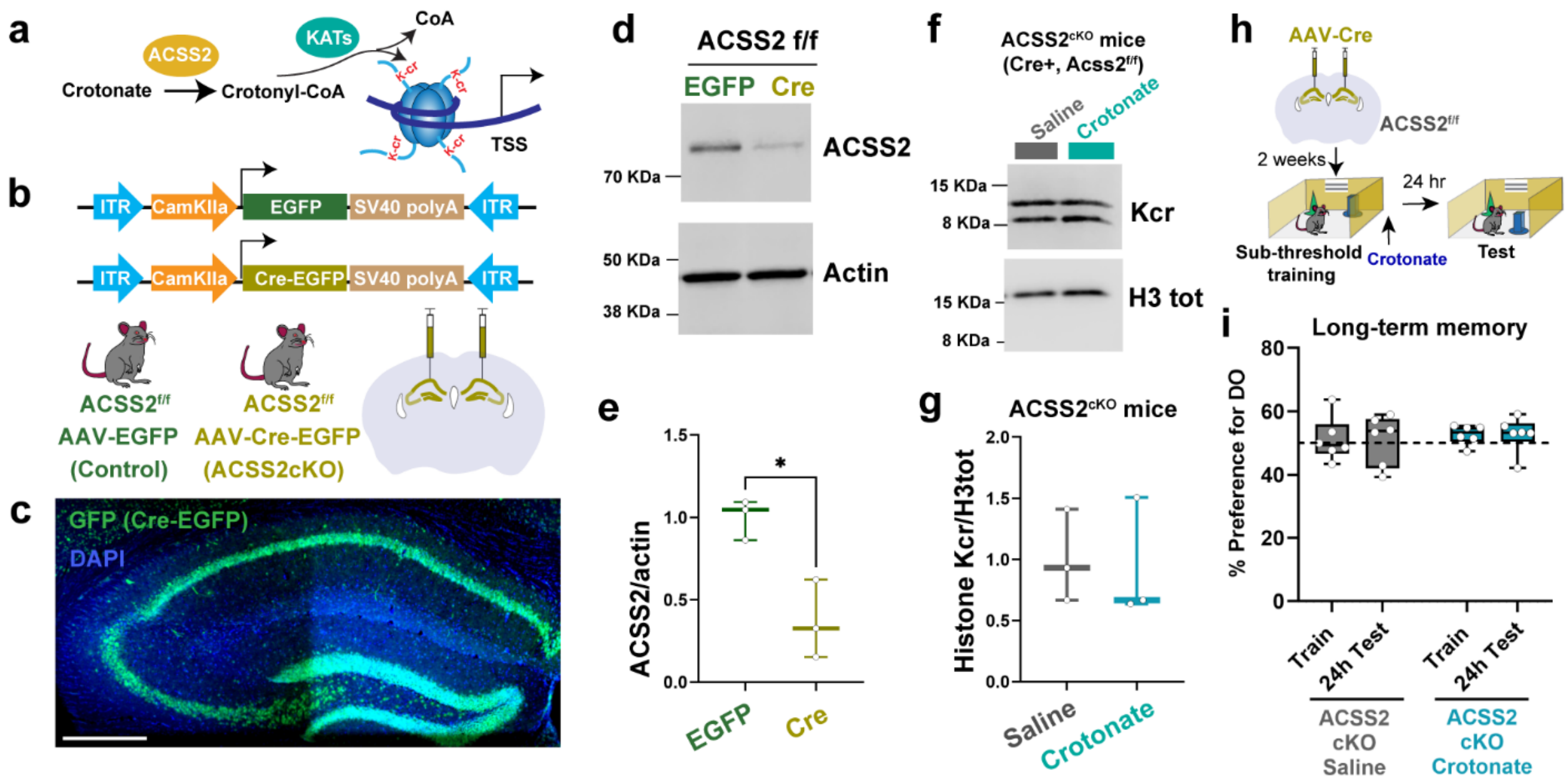

Supplementary Figure S6. Crotonate-mediated memory enhancement is dependent on ACSS2. a. Schematic of ACSS2 in regulating histone crotonylation. **b.** Schematic of viral constructs used to conditionally knock down ACSS2 in excitatory neurons of dorsal hippocampus. AAV_9-CaMKIIα-EGFP served as vector control and AAV_9-CaMKIIα-Cre-EGFP was used to drive expression of Cre recombinase in excitatory neurons of $ACSS2^{f/f}$ mice. **c.** Immunofluorescence image using GFP antibody showed expression of the Cre-EGFP in the dorsal hippocampus two weeks following viral infusion. **d-e.** Western blot from whole cell extracts of the dorsal hippocampus of $ACSS2^{f/f}$ mice infused with AAV-EGFP or AAV-Cre-EGFP. Normalized band intensity of ACSS2 across the groups: Unpaired t test: t (4) = 4.118, *p = 0.0146. EGFP (n = 3) and ACSS2 (n = 3), males only. **f-g.** Western blot using Kcr antibody from core histones extracted from the dorsal hippocampus of ACSS2 cKO mice ($ACSS2^{f/f}$ infused with AAV-Cre-EGFP) administered either crotonate (200 mg/kg, oral gavage) or saline immediately after SOR training. Normalized band intensity of pan Kcr across the groups: Unpaired t test: t (4) = 0.1853, p = 0.862. Saline (n=3), Crotonate (n=3), males only. **h-i.** Crotonate treatment after training using a sub-threshold SOR learning paradigm does not enhance long-term memory in ACSS2 cKO mice. Two-way ANOVA: no significant session x treatment interaction $F_{(1, 10)}$ = 0.0007554, p= 0.9786. Saline (n=6), Crotonate (n=6), males only. All box plots: the center line represents the median,

the box edges represent the top and bottom quartiles (25th to 75th percentiles), and the minimum and maximum whiskers

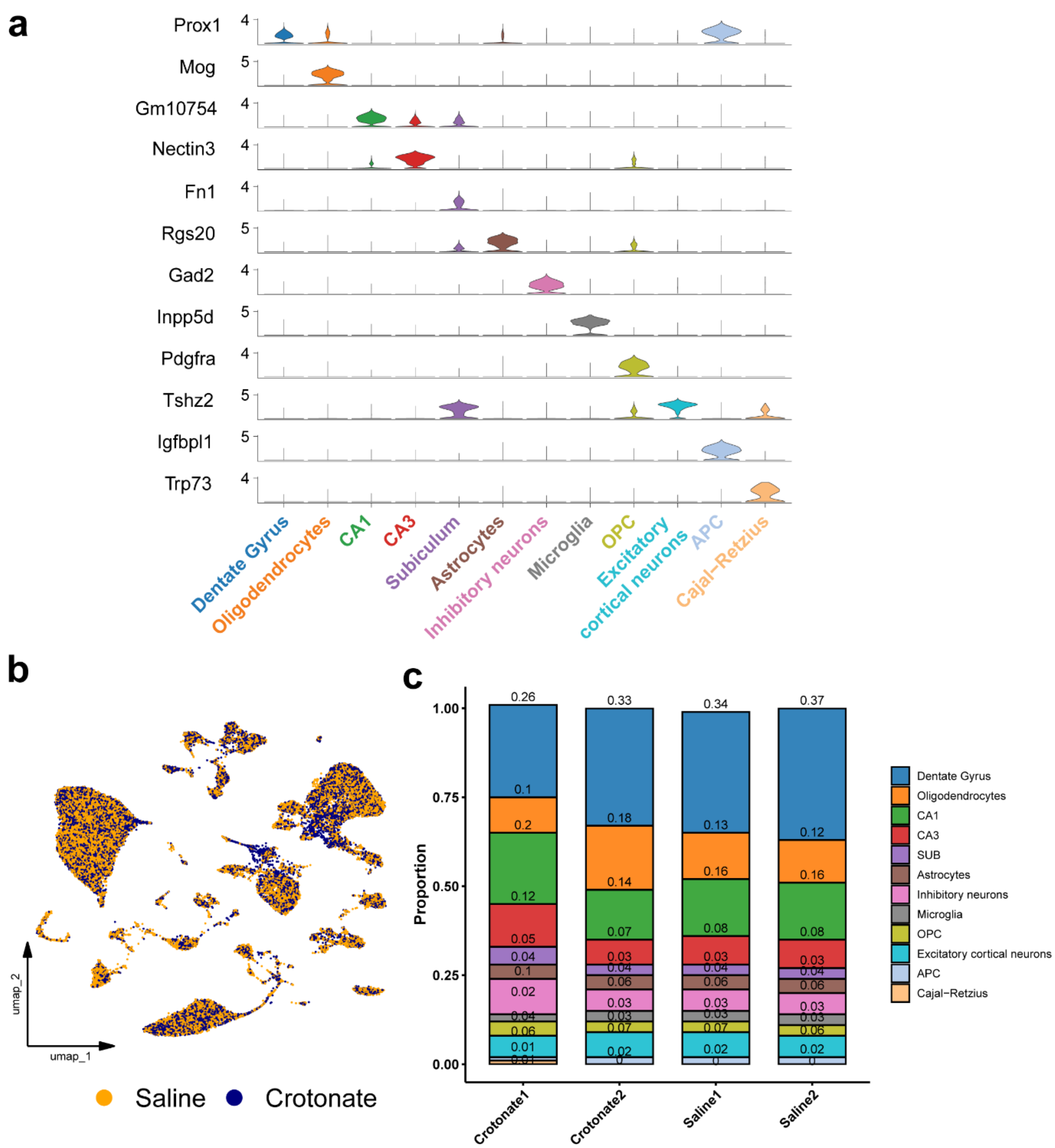

Supplementary Figure S7. a. Violin plot showing the expression profiles of marker genes across different cell types of the dorsal hippocampus. **b.** UMAP plots depicting cell-type specific clusters in saline-treated and crotonate-treated groups. **c.** Cell-type-proportions for each sample across experimental groups.

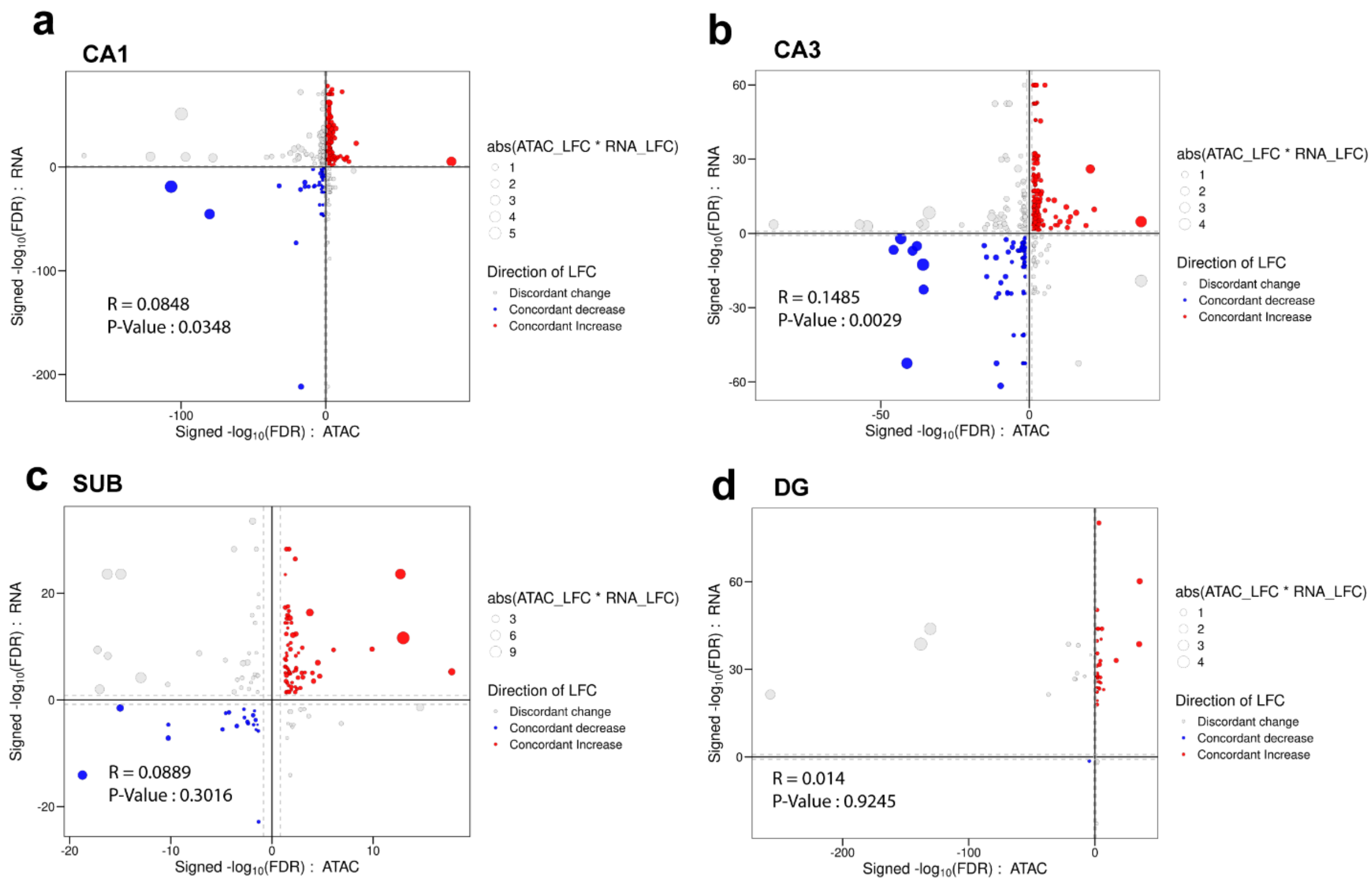

Supplementary Figure S8. Quadrant plots depicting the correlation between genes with significant differential expression and genes with significant DARs in hippocampal principal neuronal layers. a. CA1, **b.** CA3, **c.** Subiculum. **d.** Dentate gyrus (DG).

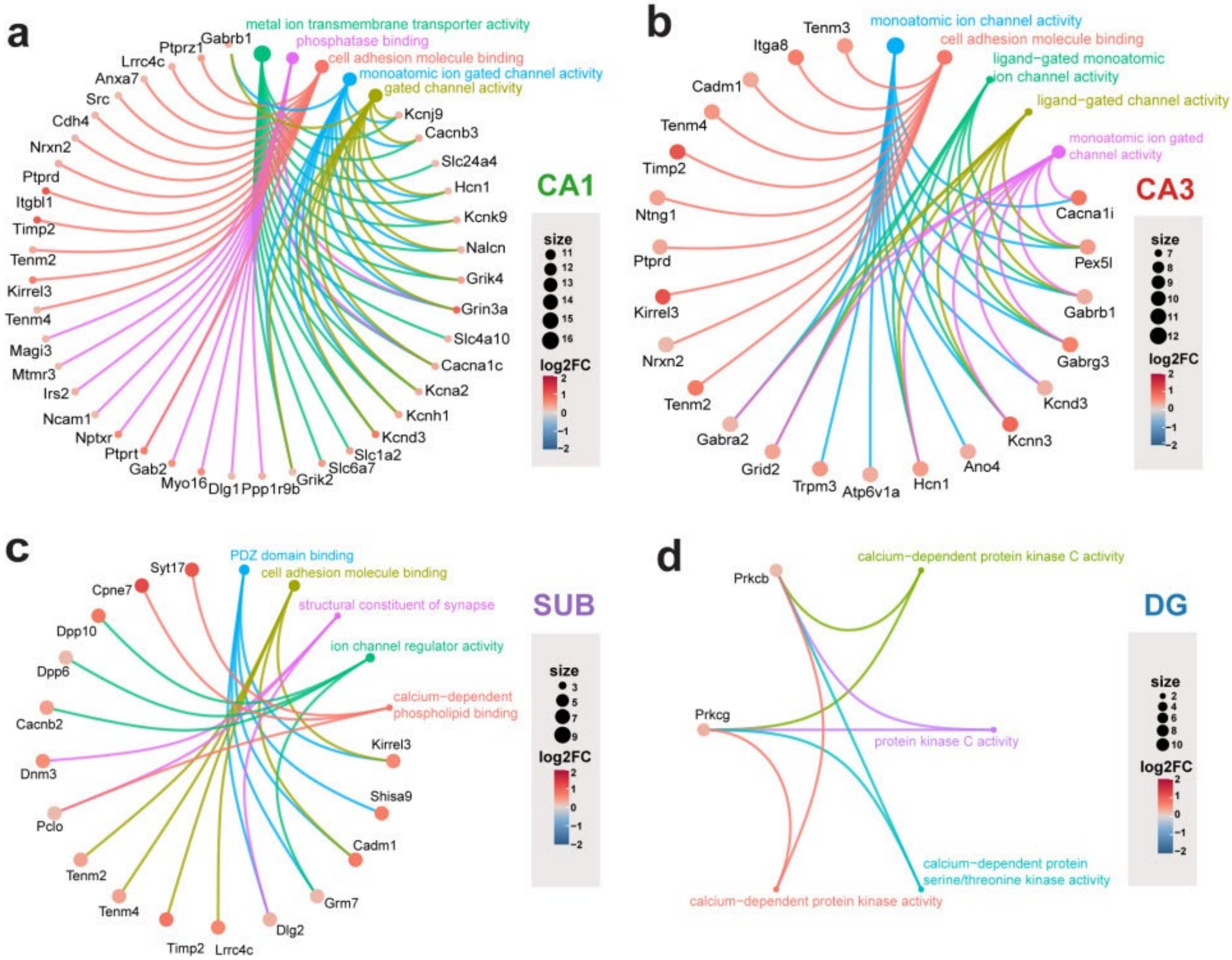

Supplementary Figure S9. Increases in Kcr elicits distinct molecular signatures in principal hippocampal neuronal cell-types. a-d. Cnet plots depicting the top five enriched pathways (GO: Molecular Function) and their corresponding DEGs with DARs in hippocampal subregions CA1 **(a)**, CA3 **(b)**, Subiculum **(c)**, and Dentate gyrus **(d)** that are upregulated in response to crotonate administration.

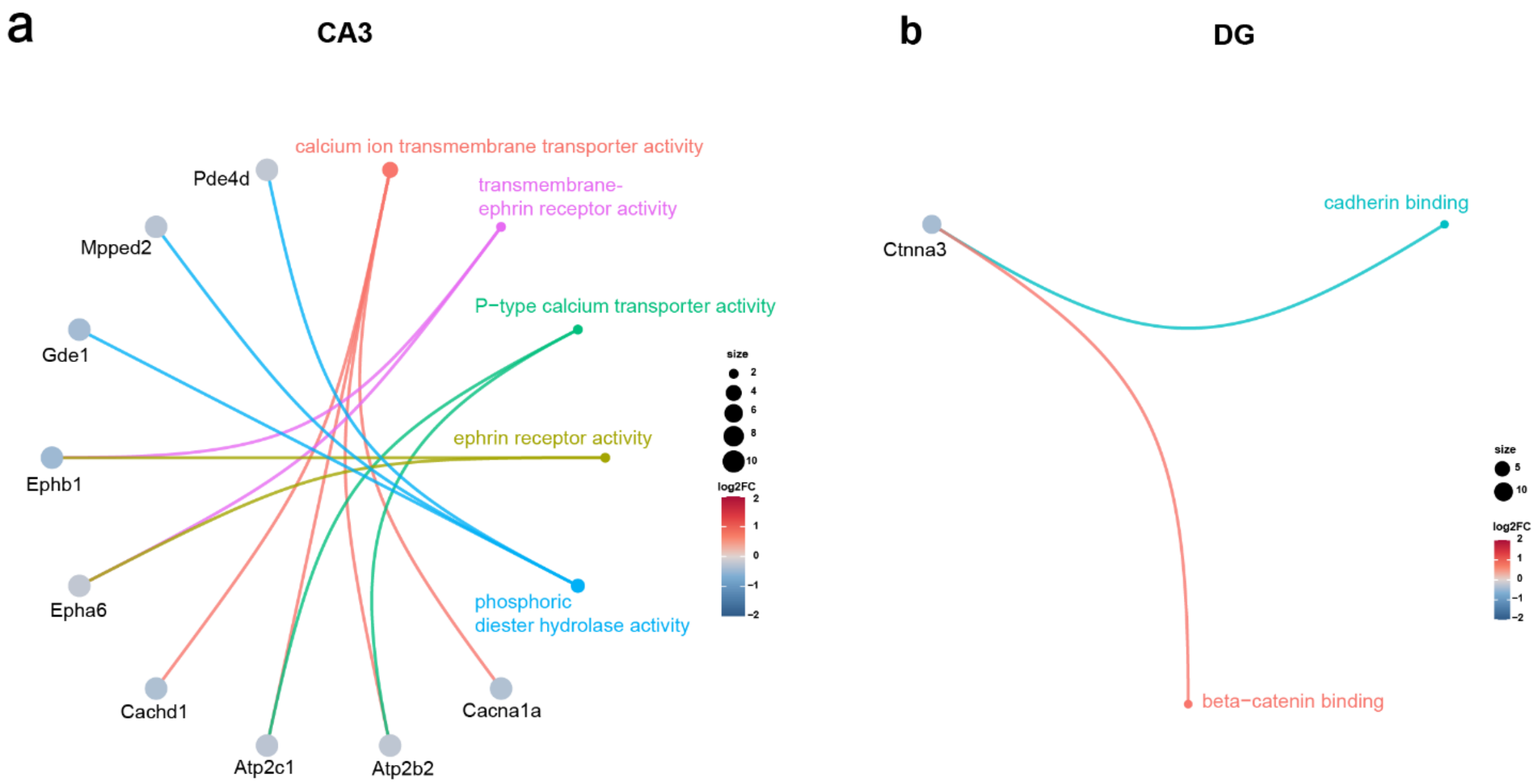

Supplementary Figure S10. Top 5 most significantly enriched GO: Molecular function patterns from downregulated genes. a. CA3. **b.** Dentate gyrus (DG).

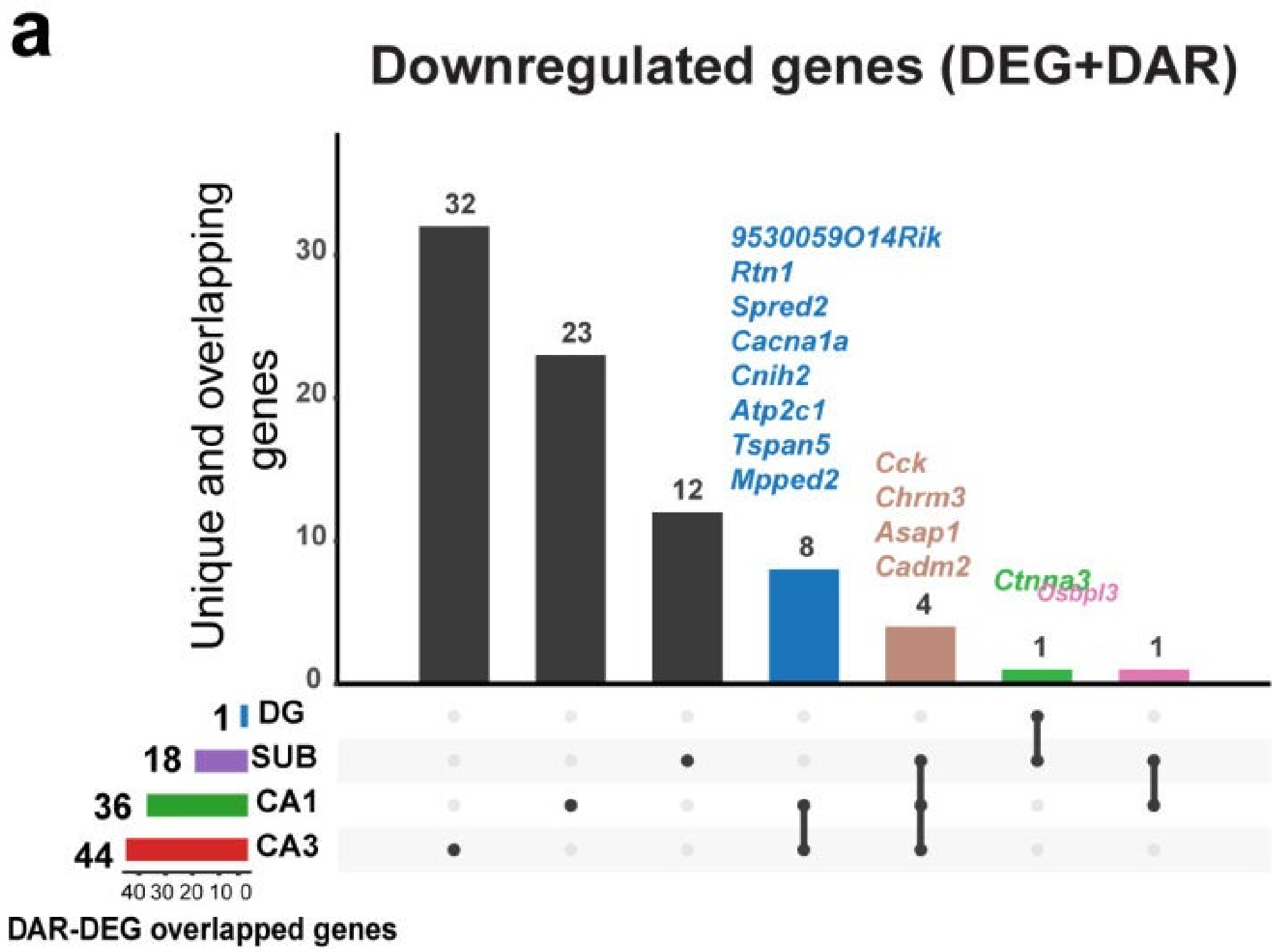

Supplementary Figure S11. a. Upset plots depicting unique and overlapping population of downregulated DEGs with DARs across all the hippocampal principal neurons.

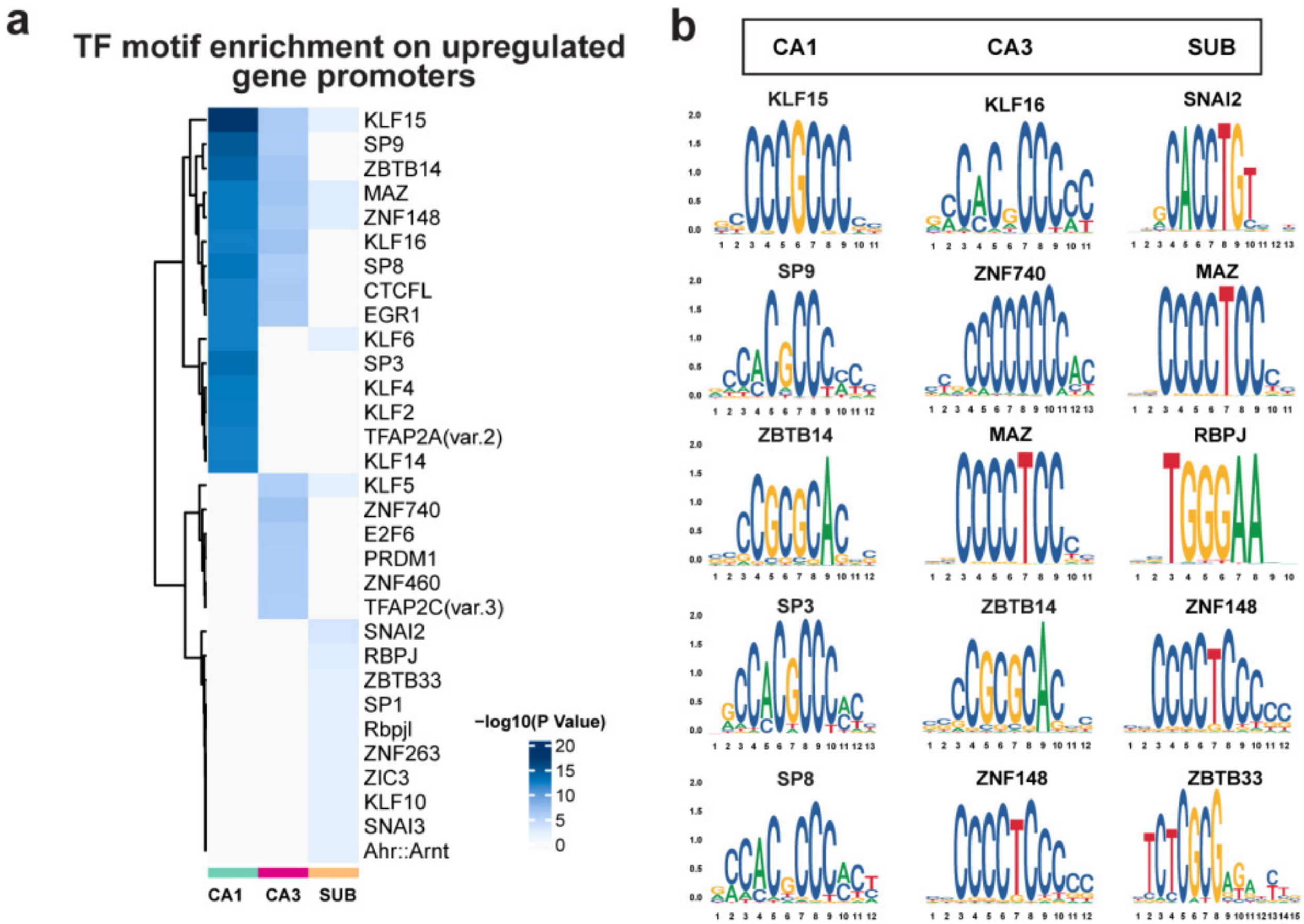

Supplementary Figure S12. Single-nuclei multiomics reveals transcription factor motif sites enriched on gene promoters that show differential expression and differential chromatin plasticity following crotonate treatment. **a.** Transcription factor (TF) motif enrichment analysis from upregulated genes with increased chromatin accessibility at the promoter upon crotonate administration. **b.** Top 5 significant TF motifs enriched from CA1, CA3, and subiculum.

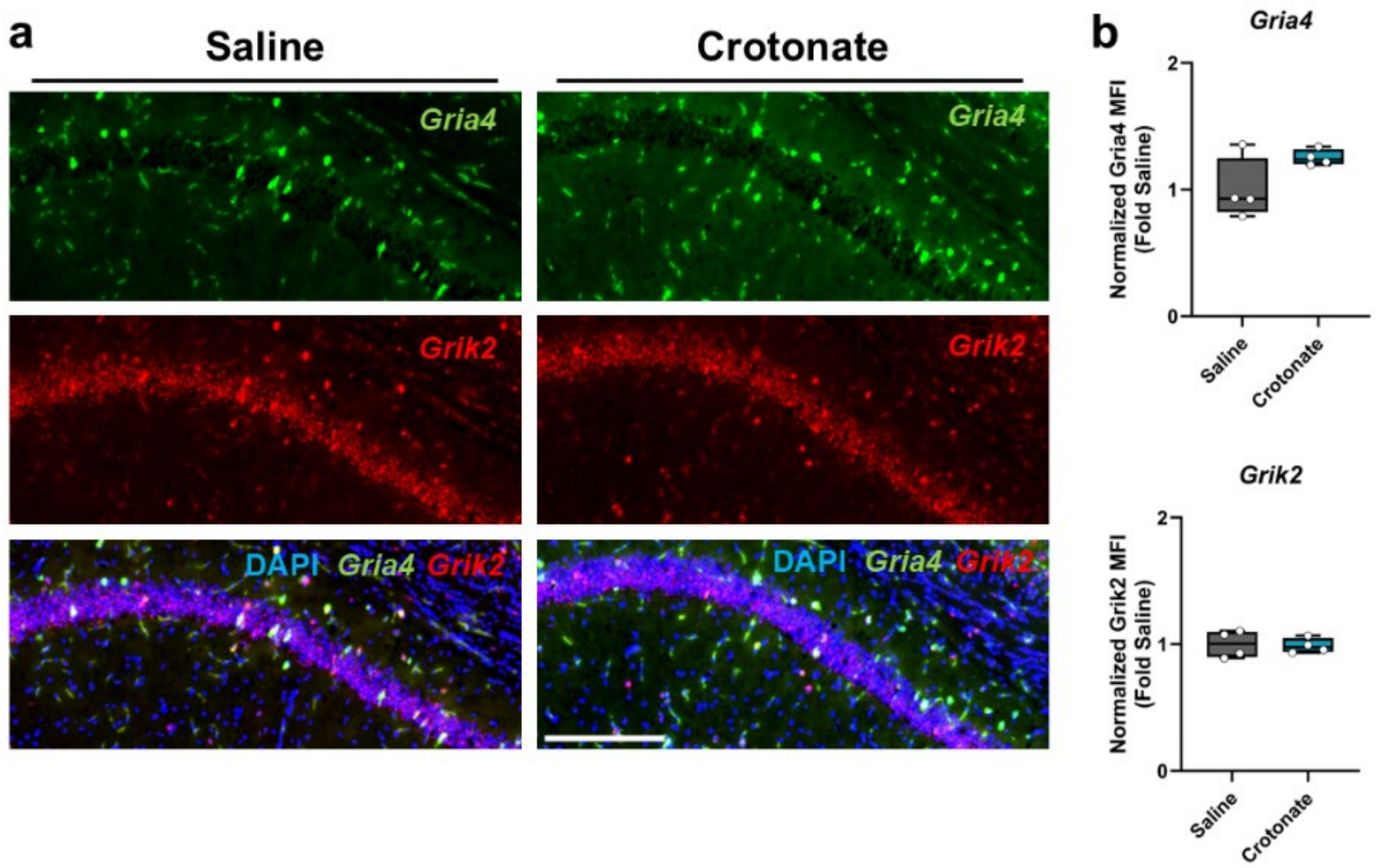

Supplementary Figure S13. Crotonate administration alone does not alter the expression of glutamatergic genes in hippocampal CA1. a. Representative *in situ* hybridization images showing the expression of *Gria4* and *Grik2* in hippocampal CA1 following crotonate or saline administration. Scalebar: 100 µm. **b.** Quantification of **a.** For *Gria4*: Normalized Mean Fluorescent Intensity (MFI) of *Gria4* levels across the groups. Unpaired t test: t (6) = 1.992, p = 0.0935. Saline (n = 4) and Crotonate (n = 4), males only. For *Grik2*: Normalized Mean Fluorescent Intensity (MFI) of *Grik2* levels across the groups. Unpaired t test: t (6) = 0.1951, p = 0.8517. Saline (n = 4) and Crotonate (n = 4), males only. All box plots: the center line represents the median, the box edges represent the top and bottom quartiles (25th to 75th percentiles), and the minimum and maximum whiskers.

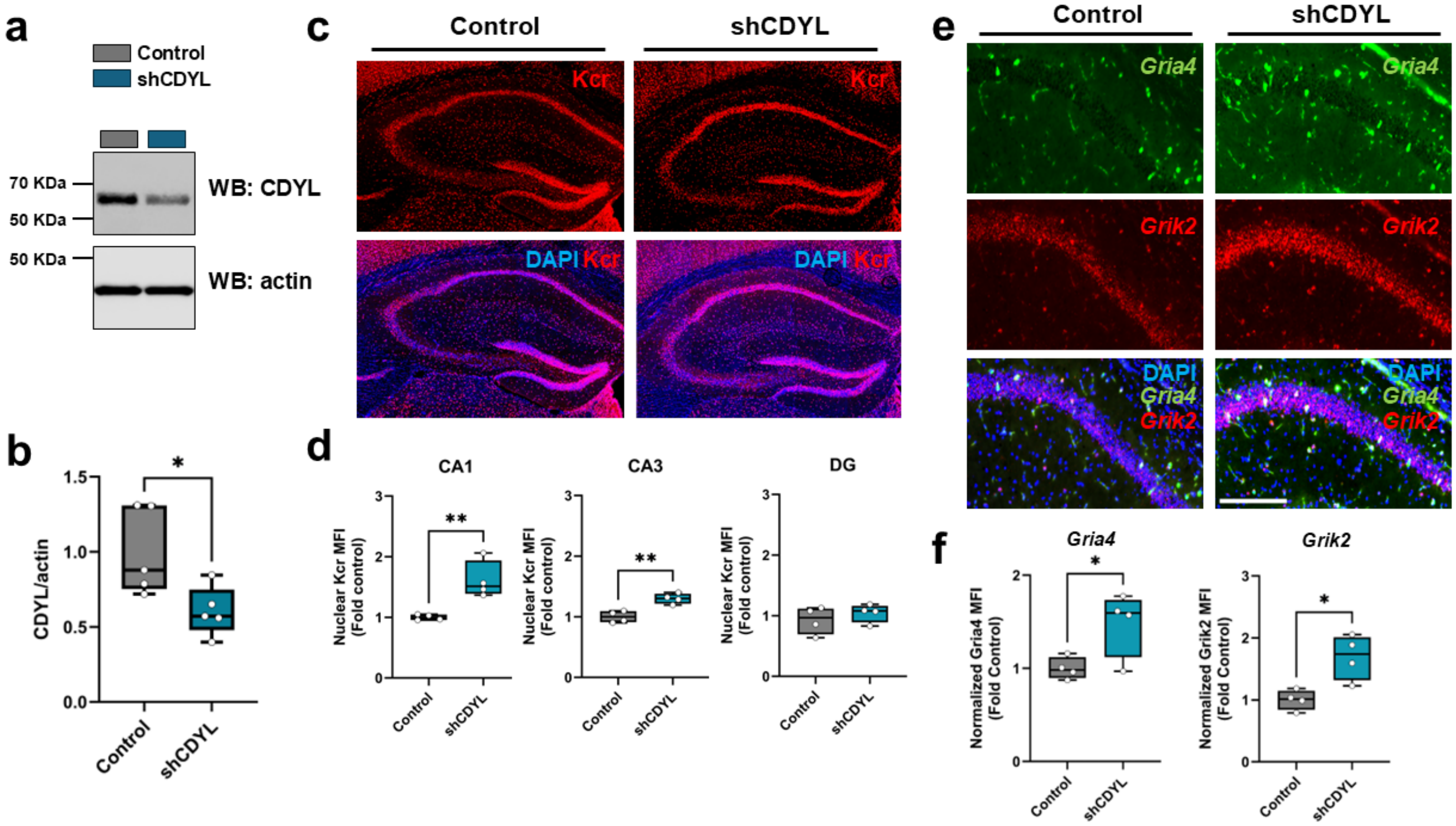

Supplementary Figure S14. CDYL KD leads to increases in hippocampal Kcr levels and in the expression of glutamatergic genes. a. Western blot showing CDYL levels analyzed from the dorsal hippocampus of mice injected with either AAV-shCDYL or AAV-scrambled (Control). **b.** Quantification of **a.** For CDYL: Normalized (with actin) CDYL expression across the groups. Unpaired t test: t (8) = 2.675, *p = 0.0281. Control (n = 5) and shCDYL (n = 5), males only. **c.** Representative immunofluorescence images depicting the hippocampal expression of pan Kcr in AAV-shCDYL and AAV-Scrambled (Control) infused mice. **d.** Quantification of **c.** For CA1: Normalized Mean Fluorescent Intensity (MFI) of Kcr levels across the groups. Unpaired t test: t (6) = 3.908, **p = 0.0079. Control (n = 4) and shCDYL (n = 4), males only. For CA3: Normalized Mean Fluorescent Intensity (MFI) of Kcr levels across the groups. Unpaired t test: t (6) = 4.652, **p = 0.0035. Control (n = 4) and shCDYL (n = 4), males only. For DG: Normalized Mean Fluorescent Intensity (MFI) of Kcr levels across the groups. Unpaired t test: t (6) = 0.8708, p = 0.4173. Control (n = 4) and shCDYL (n = 4), males only. **e.** Representative *in situ* hybridization images showing the expression of *Gria4* and *Grik2* in hippocampal CA1. Scalebar: 100 µm. **f.** Quantification of **e.** For *Gria4*: Normalized Mean Fluorescent Intensity (MFI) of *Gria4* levels across the groups. Unpaired t test: t (6) = 2.591, *p = 0.0412. Control (n = 4) and shCDYL (n = 4), males only. For *Grik2*: Normalized Mean Fluorescent Intensity (MFI) of *Grik2* levels across the groups. Unpaired t test: t (6) = 3.474, *p = 0.0132. Control (n = 4) and shCDYL (n = 4), males only. All box plots: the center line represents the median, the box edges represent the top and bottom quartiles (25th to 75th percentiles), and the minimum and maximum whiskers.

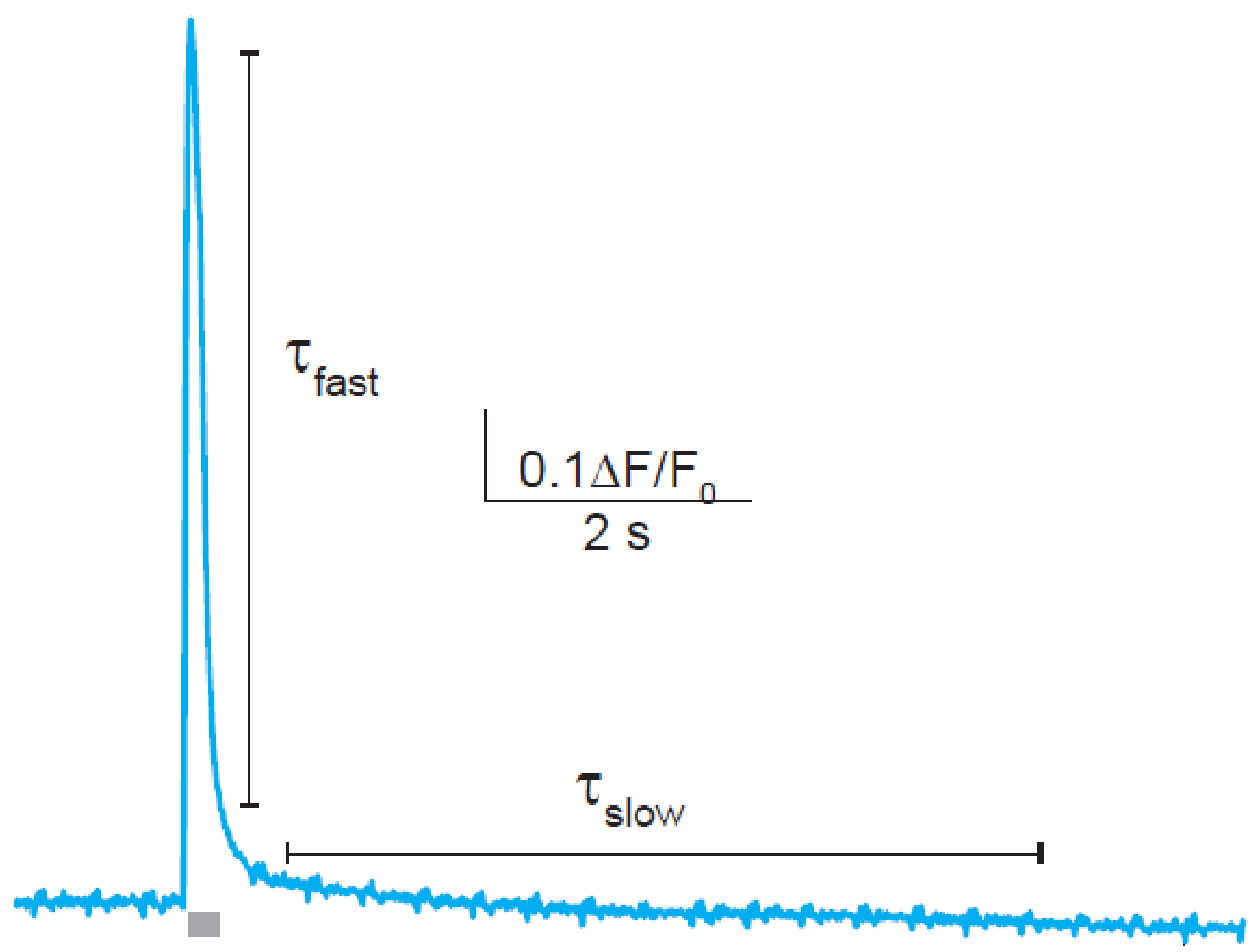

Supplementary Figure S15. Computation of glutamate clearance rates. Glutamate clearance kinetics were determined by fitting the decay of the glutamate response with two single-term exponential equations (black solid line); blue, glutamate fluorescence from a single trial; τ_{fast}: stimulus offset to ~4s, τ_{slow}: ~4s to 10s.

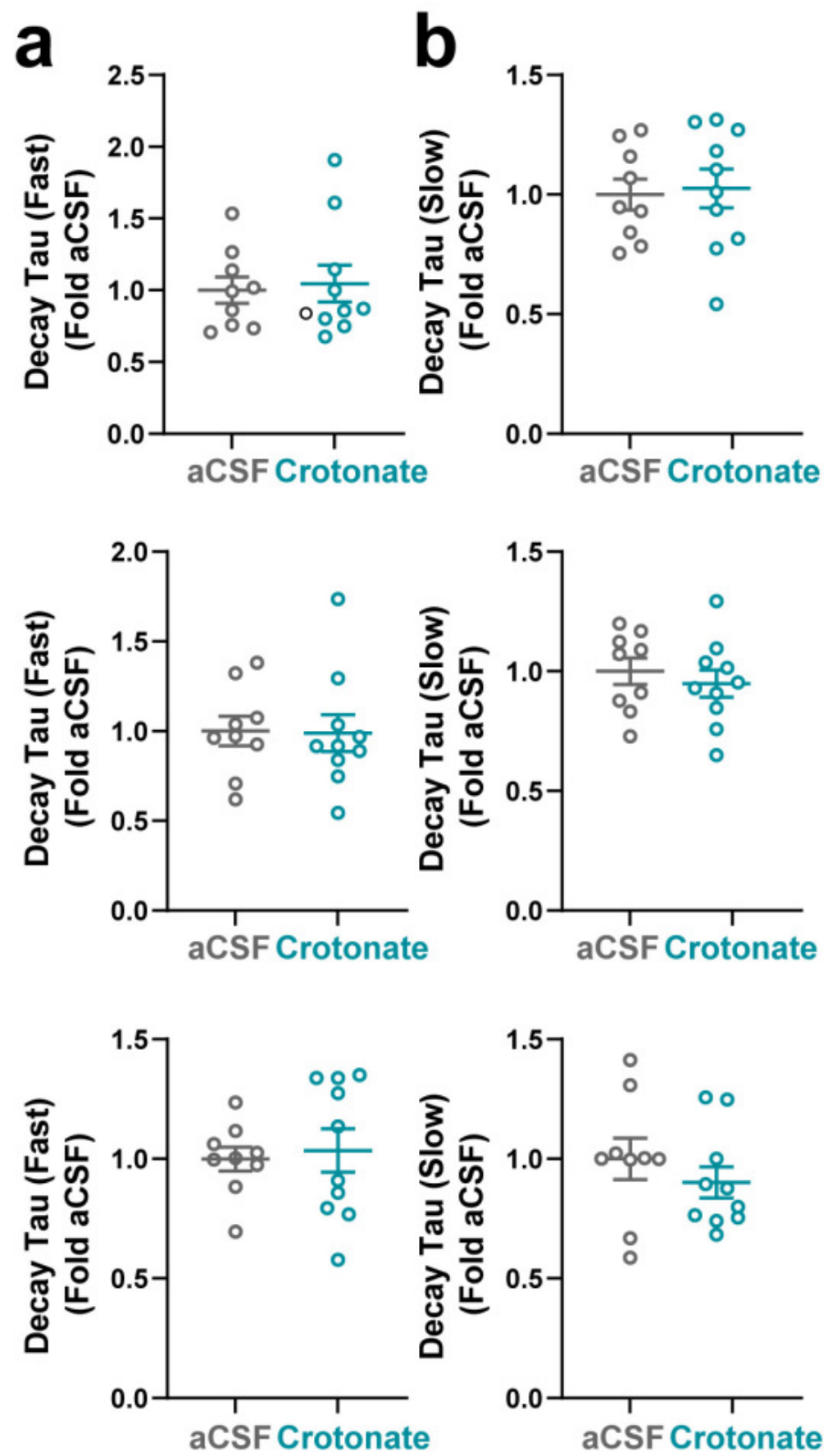

Supplementary Figure S16. Crotonate-dependent increases in Kcr do not affect glutamate decay kinetics. a. No differences in fast decay kinetics were observed between the two groups across all stimulation frequencies. For 10 Hz (top): Lognormal Welch's t test: t (16.74) = 0.1425, p = 0.884. aCSF (n = 9) and Crotonate (n = 10). For 20 Hz (middle): Lognormal Welch's t test: t (16.94) = 0.2031, p = 0.8415. aCSF (n = 9) and Crotonate (n = 10). For 50 Hz (bottom): Welch's t test: t (13.89) = 0.3411, p = 0.7381. aCSF (n = 9) and Crotonate (n = 10). **b**. No differences in slow decay kinetics were observed between the two groups across all stimulation frequencies. For 10 Hz (top): Lognormal Welch's t test: t (15.98) = 0.0768, p = 0.9397. aCSF (n = 9) and Crotonate (n = 10). For 20 Hz (middle): Welch's t test: t (16.98) = 0.6512, p = 0.5236. aCSF (n = 9) and Crotonate (n = 10). For 50 Hz (bottom): Lognormal Welch's t test: t (14.94) = 0.7948, p = 0.4392. aCSF (n = 9) and Crotonate (n = 10). All scatter plots: the center line represents the mean, error bars represent ±SEM.

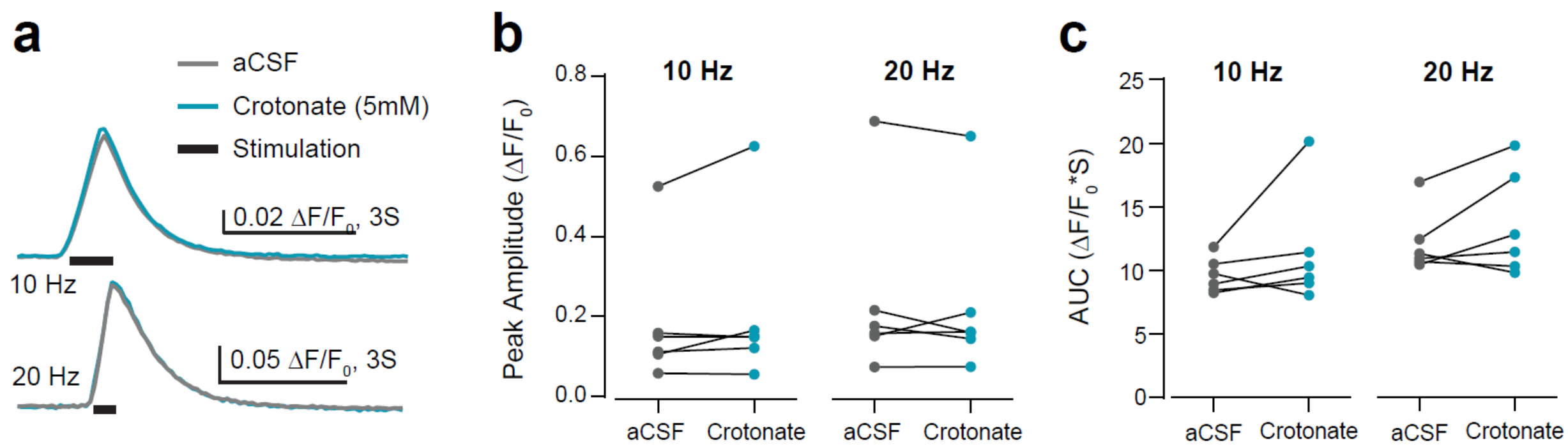

Supplementary Figure S17. Examining non-genomic effects of crotonate. Brief incubation with crotonate (5mM) does not exert any effect on the peak amplitude (**b**) or AUC (**c**) of stimulus-evoked calcium transients. **b.** Peak amplitude: For 10 Hz (7): Paired t test: t (5) = 1.446, p = 0.2077. For 20 Hz (middle): Paired t test: t (5) = 0.6143, p = 0.5659. **c.** Area under the curve (AUC): For 10 Hz (7): Paired t test: t (5) = 1.290, p = 0.2534. For 20 Hz (middle): Paired t test: t (5) = 1.522, p = 0.1886. aCSF (n = 6) and Crotonate (n = 6).

Supplementary Table Legends

Dataset S1: Comparison of the proportion of cell-type clusters identified between crotonate- and saline-treated groups.

Dataset S2: Merged dataset of differential changes in cell-type-specific gene expression and chromatin accessibility in crotonate-treated group compared to the saline-treated group.

Dataset S3: Gene Ontology (Cellular Component) overrepresentation analysis of the significant DEGs that exhibit significant differential chromatin accessibility in hippocampal cell-types CA1 and CA3.

Dataset S4: Gene Ontology (Molecular Function) overrepresentation analysis of the significant DEGs that exhibit significant differential chromatin accessibility in hippocampal cell-types CA1, CA3, DG, and Subiculum.

Dataset S5: Transcription Factor Motif enrichment analysis performed on the upregulated genes in CA1, CA3, and subiculum clusters that exhibit increased chromatin accessibility in their promoter regions (TSS±2kb) following crotonate administration.
